# First forelimb reconstruction and range of motion assessment of the Late Cretaceous dinosaur *Troodon formosus*

**DOI:** 10.7717/peerj.20987

**Published:** 2026-07-16

**Authors:** Michael Serio, Ashley Heers, David Varricchio, Han Hu

**Affiliations:** 1 Key Laboratory of Vertebrate Evolution and Human Origins, Institute of Vertebrate Paleontology and Paleoanthropology, Chinese Academy of Sciences, Beijing, China; 2 University of Chinese Academy of Sciences, Beijing, China; 3 Department of Earth Science, Montana State University, Bozeman, MT, United States of America; 4 Scripps College, Claremont, CA, United States of America

**Keywords:** Biomechanics, 3D reconstrucion, Range of motion, *Troodon formosus*, Fossil digitization, 3D modeling

## Abstract

*Troodon formosus* (*T. formosus*) is a theropod dinosaur from the Late Cretaceous of North America. *T. formosus*, like many theropods, are speculated to have used their forelimbs to hunt, and the complexity of their preserved egg clutches suggests *T. formosus* may have also used its forelimbs to move its eggs. Understanding the morphology and range of movement of *T. formosus*’s forelimb could help shed light onto these hypotheses. However, no complete forelimb material has yet been found for *T. formosus*, and a 3D reconstruction and range of motion (ROM) estimate has not been attempted. This study aims to address this gap by leveraging digital modelling technology to create the first forelimb reconstruction and ROM for *T. formosus*. Surface scans from multiple *T. formosus* fossils housed in the Museum of the Rockies (Bozeman, Montana) were digitally combined to reconstruct a nearly complete forelimb. Digital articulations based on this assembled model were compared with physical ROM using 3D printed copies. Results show higher ranges of flexion than extension in *T. formosus*’s joints, consistent with closely related species. However, *T. formosus* shows higher manual extension than close relatives. The humerus also shows anatomy convergent with more basal theropod species. These differences may imply a divergent morphology and function of the manus, as well as a deviation from avian ancestor forelimb morphology. ROM results cannot confirm whether *T. formosus* was able to grasp objects single-handed, but two-handed apprehension of objects, including eggs, remains feasible.

## Introduction

*Troodon formosus* (*T. formosus*), described in 1856 from an isolated tooth, was initially classified as a lacertilian reptile, then reassigned to various dinosaur families before being recognized as a small theropod in 1945 (Leidy, 1860; Nopcsa, 1901; Gilmore, 1924; Sternberg, 1945). *T. formosus’s* validity is debated, with proposals to either synonymize it with *Stenonychosaurus inequalis* (*S. inequalis*), or to establish *T. formosus* as the senior synonym (Van der Reest & Currie, 2017; Varricchio, Hogan & Gardner, 2025). For the purposes of this project, all troodontid material collected from the uppermost member of the Two Medicine formation is assigned to *T. formosus* as the senior synonym of *S. inequalis* (Varricchio, Hogan & Gardner, 2025).

In the 1990’s, new material for *T. formosus* was discovered in The Two Medicine Formation, dated contemporaneously with the Judith River Formation (Rogers, Swisher & Horner, 1993; Ramezani et al., 2022). Most specimens come from Jack’s Birthday Site (JBS), a Late Cretaceous shallow floodplain deposit, dated to just older than 75.25 Ma (Varricchio, 1995; Rogers et al., 2025). JBS has yielded multiple hadrosaur species, tyrannosaurids, and *T. formosus* material, including the only recorded multi-individual occurrence of any troodontid. In 1993, *T. formosus* nests were discovered, confirming some nesting behaviors shared with birds (Varricchio et al., 1997). Evidence suggests nesting fidelity at nest sites, and clutch shape indicates the manipulation of eggs post-laying (Varricchio, Jin & Jackson, 2015). Gastric pellets bearing mammalian skeletal material have also been attributed to *T. formosus* (Freimuth et al., 2021). These findings support the image of *T. formosus* as an agile small game hunter with advanced eyesight and near avian intelligence (Russell, 1969; Varricchio, Hogan & Freimuth, 2021).

**Figure 1:**
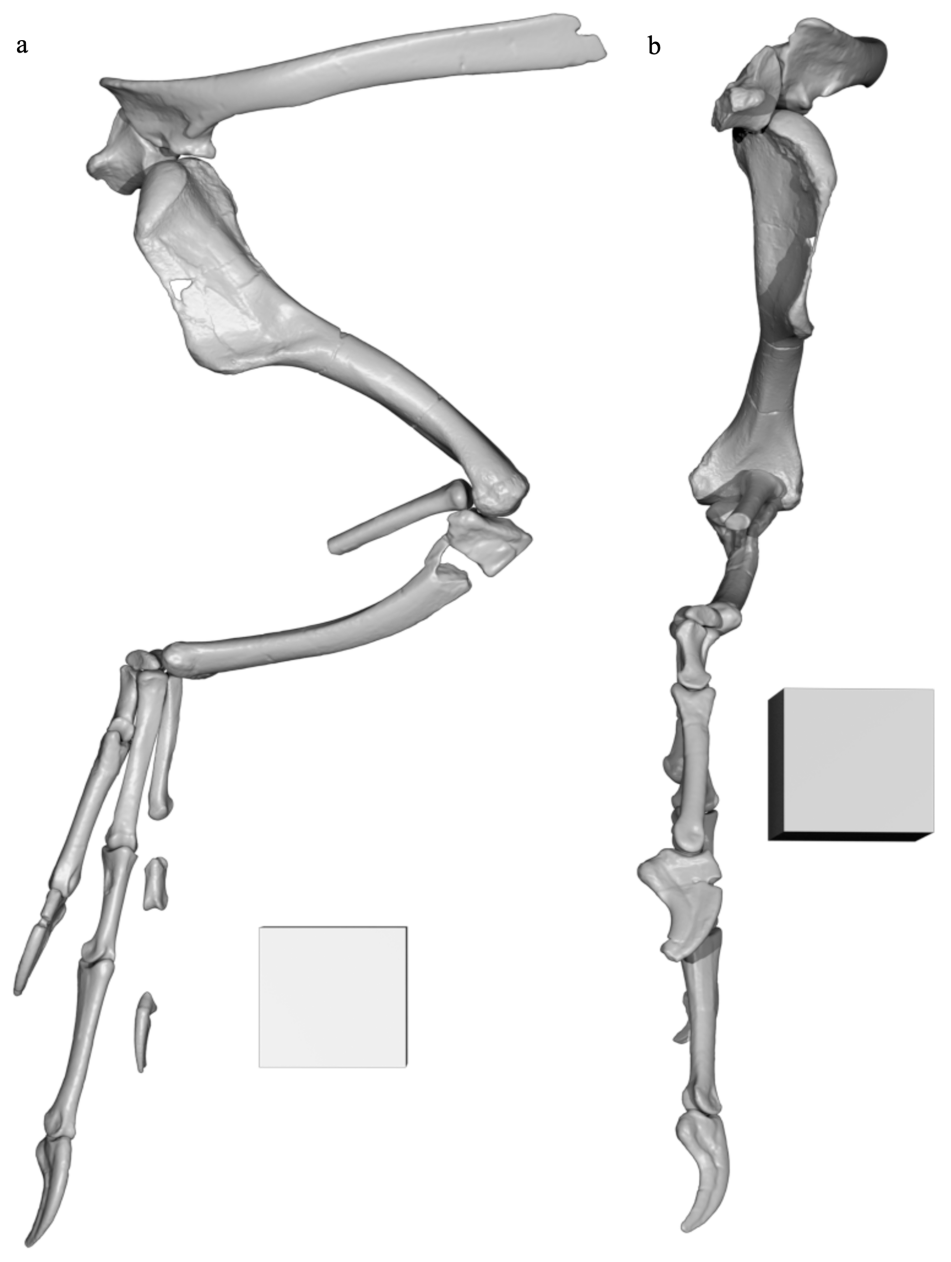
Realistic forelimb pose for *Troodon* in (A) lateral and (B) anterior view. Limb orientation based on work by Senter & Robins (2015) and aligned using the range of motion determined from this study. Scale cube is five cm.

Considering these inferences, *T. formosus* is hypothesized to have had dexterous hands and mobile arms (Russell & Seguin, 1982). Previously, a reconstruction was unfeasible due to incomplete skeletal material, but advances in surface scanning and digital modeling have allowed for the reconstruction of a complete forelimb (Fig. 1). This new reconstruction enables a range of motion (ROM) assessment and the testing of old hypotheses, especially those established by Senter (2006a), which include the ability to grab and hold objects, especially eggs, either between two hands, to the chest, or single handedly.

From this reconstruction, ROM at each joint was digitally measured. ROM studies have long focused on fossil bones or casts being physically manipulated to assess joint mobility (Galton, 1971; Sereno, 1994; Novas & Puerta, 1997; Carpenter, 2002; Senter, 2006a; White et al., 2015; Senter & Sullivan, 2019). These studies typically exclude soft tissues, like cartilage, and assume the maximum mobility of a joint is bone-on-bone contact where the articulated surface of the distal bone will not pass the extent of the articulated surface of the proximal bone (Senter & Sullivan, 2019; Yu et al., 2022). These studies are less adept at revealing whether a position would be adopted by an organism but excel at identifying unrealistic positions. Numerous ROM studies have focused on theropod genera, with a small subset concerned with forearm ROM, and even fewer on paravian forearms (Carpenter & Smith, 2001; Carpenter, 2002; Senter & Robins, 2005; Senter, 2006b; Senter, 2006a; White et al., 2015; Yu, Corwin & Xing, 2015; Senter & Sullivan, 2019; Yu et al., 2022). These studies reveal a trend in the evolution of forelimb mobility, with basal theropods exhibiting higher degrees of extension compared to flexion, and species closer to Avialae showing higher degrees of flexion over extension (Yu et al., 2022).

Digital ROM studies have advanced over the last two decades (Mallison, 2010; Baier & Gatesy, 2013; Manafzadeh & Padian, 2018; Herbst, Manafzadeh & Hutchinson, 2022), with some methods of adding soft tissue reconstruction (Burch, 2014; Heers, Rankin & Hutchinson, 2018; Bishop, Cuff & Hutchinson, 2021). X-ray analysis of moving joints, also known as X-ray Reconstruction of Moving Morphology (XROMM), (Gatesy et al., 2010; Baier & Gatesy, 2013), has confirmed joint movement in up to six degrees of freedom, with three rotational (flexion-extension, abduction-adduction, and long-axis rotation) and three translational (medial-lateral translation, anterior-posterior translation, and distraction-compression (dorsoventral) translation) movements (Kambic, Roberts & Gatesy, 2014; Manafzadeh & Gatesy, 2021). XROMM has also revealed that small changes in long-axis rotation significantly affect joint function. Applying this to extinct taxa has remained challenging, though recent efforts are addressing this gap (Bishop, Cuff & Hutchinson, 2021; Manafzadeh & Gatesy, 2022; Manafzadeh, Gatesy & Bhullar, 2024). Most digital ROM studies on extinct taxa have focused on the hindlimb (Manafzadeh & Padian, 2018; Bishop, Cuff & Hutchinson, 2021; Manafzadeh, Gatesy & Bhullar, 2024). Newer studies are starting to apply this newer methodology to the forelimb (Demuth, Rayfield & Hutchinson, 2020; Richards et al., 2021; Bishop, Brocklehurst & Pierce, 2023; Griffin et al., 2024; Brocklehurst et al., 2025); however, dinosaur forelimb studies have retained the traditional bone-on-bone approach (White et al., 2015; Senter & Sullivan, 2019).

This study, while applying much of this newer methodology, is not an exhaustive application of these techniques. Instead, it leverages digital techniques in a manner more comparable to traditional physical ROM studies, allowing ROM testing for the first time in *T. formosus’s* forelimb. This includes testing hypotheses on egg grasping ability for nest construction, general forearm functionality, potential hunting behavior and comparing the ROM of *T. formosus* and other theropods.

## Materials & Methods

### Materials

Although cranial, axial, and appendicular skeletal elements for *T. formosus* have been recovered, no complete skeleton has yet been found. Articulated material is rather rare, and most collected specimens are isolated skeletal elements. Fossils in this study collected from JBS show minimal crushing, with breakage primarily perpendicular the length of the element, indicating damage that occurred post fossilization. Additional material used for this study was found from the Blacktail Creek locality within the Two Medicine Formation (MOR locality TM-071, museum number MOR 563). Specimens referenced from Blacktail Creek include nearly all forelimb elements, and are from a partially articulated, incomplete individual, but are more corroded relative to JBS material. Both localities occur in close stratigraphic proximity within the upper portion of the Flag Butte Member of the formation (Rogers et al., 2025).

While incomplete, there is enough material collected from the Two Medicine sites to represent most of a forelimb. The only bones missing from the reconstruction include two phalangeal bones from the third digit (III) and the radiale. In total there were sixteen bones used, representative of at least four individuals. All the bones in this study are accessioned at the Museum of the Rockies (MOR, Bozeman, MT). Most of the elements used were from MOR 553 S and MOR 553 L (museum number MOR 553), representing multi-species assemblages collected from JBS, and additional elements from MOR 563, an associated skeleton collected from Blacktail Creek. For detailed information on the elements in this study, refer to the Supplemental Information.

**Scapula**. The scapulae used from MOR 553 has a thin medially curving blade showing no signs of taphonomic alteration. The distal end is broken, but proximally the glenoid fossa, acromial process, and coracoid suture are preserved.

**Coracoid**. The MOR 553 coracoid preserves the complete glenoid fossa and scapular suture, but is incomplete medially, including the dorsal part of the acromial process. Together, the coracoid and scapula preserve the full glenoid fossa allowing shoulder joint analysis.

**Humerus**. The reconstruction used two robust humeri from MOR 553, which exhibit finished cortical exteriors and well-defined articular surfaces, indicative of maturity.

**Ulna**. The ulna was nearly complete but broken at its proximal end. Its fragments aligned seamlessly, with no distortion visible, and were digitally merged in Maya to restore the bone’s original length.

**Radius**. The radius used from MOR 553 preserves the complete proximal surface, and a short section of the shaft in good condition. There is a radius from MOR 563 that preserves an abraded section of the midshaft, so only the MOR 553 radius was used in the final reconstruction.

**Semilunate Carpal**. The MOR 553 semilunate carpal is well preserved, with detailed proximal and distal articular surfaces. It fits tightly with metacarpals I and II. A small convex facet likely represents an unfused distal carpal 4, indicating immaturity (Xu, Han & Zhao, 2014).

**Metacarpals I-III**. The MOR 553 metacarpals are complete and minimally abraded, while those from MOR 563 are incomplete. Given JBS quarry material, MOR 553 elements likely represent separate individuals.

**Phalanx I-1**. Phalanx I-1 is a composite of the proximal half from MOR 563 and distal half from MOR 553. This replaced the incomplete MOR 553 proximal facet without affecting overall length (see Supplemental Information).

**Ungual Phalanx I-2**. The MOR 553 first ungual is the largest ungual, typical of theropods. It is thin in profile, tapering distally, with the tip broken off.

**Phalanx II-1**. The MOR 553 S digit first phalanx is complete, with only minor dorsal crushing.

**Phalanx II-2**. The second digit second phalanx is the longest of the phalanx bones. It is broken in two places, but otherwise well-preserved with clear joint surfaces.

**Ungual Phalanx II-3**. The MOR 553 S ungual is smaller than the first. When viewed laterally it has a faintly tapering teardrop shape.

**Phalanx III-2**. The MOR 553 Phalanx III-2 is the only non-ungual phalanx preserved for digit III. It is much shorter than other phalanges and features a prominent proximal facet, like other theropods.

**Ungual Phalanx III-4**. The last MOR 553 ungual phalanx is the smallest and most partial. The distal end and flexor tubercle are broken, though the dorsal curvature and part of the proximal facet are visible.

### Bone scanning

Eighteen forelimb elements were scanned at MOR using a Creaform GO! Surface 3D scanner (AMETEK Inc.) at a 0.30 mm resolution. Two scans, a distal and proximal of each element, were initially made and then both were combined into one digital element. The single element was cleaned using VXelements 3D software and saved as a .STL file. Completed .STL files were then imported into the 3D animation software, Maya (Autodesk) and converted to .OBJ format. To complete the left side material, right sided forelimb elements, including the scapula, coracoid, humerus, and metacarpal-I, were mirrored. The selected digital elements were then scaled to form a single left forelimb, as detailed below.

### Scaling

The eighteen bones scanned for this project were unassociated and assumed to come from different individuals. To address size variation, all elements except the humerus and unguals were scaled to better represent one individual. *T. formosus* hindlimb bones show mixed isometric and allometric growth, and a comparison between humeral and femoral lengths of Troodontidae show negative forelimb allometry relative to body size (Palma Liberona et al., 2019; Boekenheide, 2023). However, limited forelimb material prevents assessing the effects of ontogeny on allometry within the *T. formosus* forelimb. Considering this gap, isometric scaling was assumed for the sake of a consistent and simple model (see Supplemental Information). Physical measurements of the *T. formosus* forelimb were verified digitally using the measure tool in Maya. JBS specimens (MOR 553) were treated as if they were from different individuals, while associated but poorly preserved MOR 563 material was assumed to be a single individual and served as a reference for scaling the MOR 553 elements.

Scaling was based on MOR 563 humeral proportions, calculated by dividing each forelimb element’s length by the MOR 563 humerus length. These ratios were multiplied by the length of a mature MOR 553 humerus to estimate adult dimensions. The MOR 553 humerus was chosen for completion, size and preservation of the full deltopectoral crest.

The above scaling method was applied to the ulna and the available carpals. When maximum lengths were missing from MOR 553 or 563, specimen scaling was adjusted using maximum widths of the specimen, or other methods to approximate the greatest length. A summary of the scaling approach for each element can be found in the Supplemental Information.

### Additional modification

After scaling, several elements required further modification. Phalanx I-1 was reconstructed by splicing complete proximal and distal portions from MOR 563 and 553, respectively. The ulna’s two broken fragments were digitally merged to restore full length, and the ulna and radius were combined using Maya’s ‘Combine’ command to simplify joint construction. The scapula and coracoid were likewise combined. Finally, the humeral distal condyle’s articular surface was refined using a more complete MOR 553 specimen in Maya. The spliced and modified elements reduced the original 18 elements to 16, with only the coracoid, radius, and ulna incomplete in the final reconstruction.

### Joint construction

Despite being incomplete, several joint surfaces were preserved and could be analyzed. A proximal humerus and glenoid fossa on the coracoid were preserved, allowing for the analysis of the shoulder joint. The distal humerus, and proximal radius and ulna were also preserved, allowing the elbow joint to be analyzed. The missing distal radius, radiale, and phalanges III-1 and III-3 prevented assessment of wrist mobility and digit III movement.

The finalized digital reconstruction created in Maya is representative of a nearly complete scaled left adult forelimb of *T. formosus* (Fig. 1). Once finalized, each digital bone was printed using a Formlabs Form 2 resin 3D printer at a 0.30 mm resolution, the same resolution with which the fossil bones were scanned (Formlabs, Somerville, MA). The 3D printed resin copies represent a 1:1 physical copy to the digital model. These physical counterparts to the digital reconstruction were used as a reference when designing and implementing the digital joint reconstruction. As the digital joint system was created, it was checked against the physical copies to confirm realistic joint movement and location.

**Figure 2:**
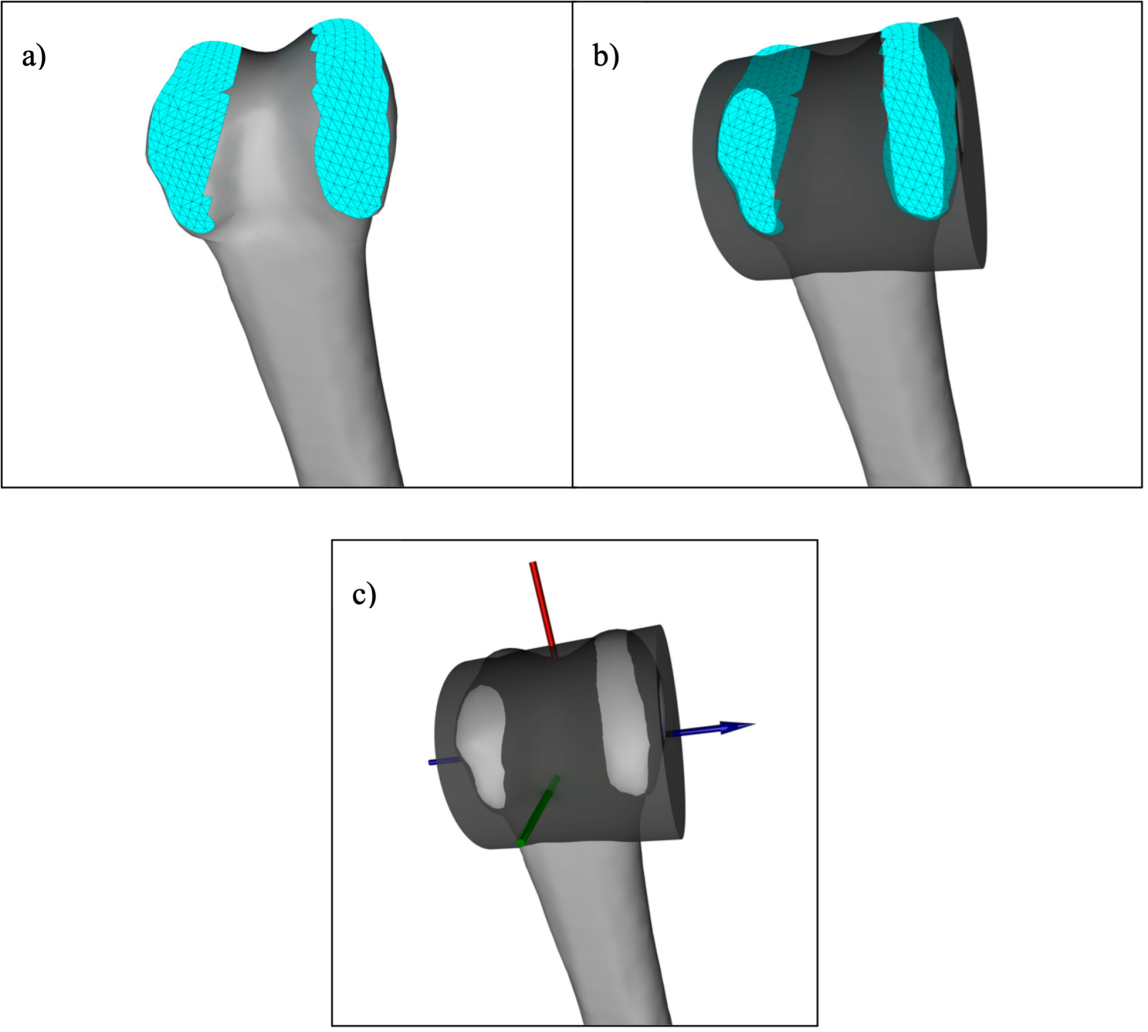
Step by step process for creating an anatomical coordinate system (ACS). (A) Selection of articulated surfaces. (B) Best fitting cylinders to selected shape. (C) Creation of XYZ axes at cylinder center.

**Shape fitting articular surfaces**. After import and modification in Maya, each digital bone was imported into Geomagic Wrap 3D mesh software (Artec3D, Luxembourg) to generate articular surfaces for each joint. First, the articulated surfaces of each bone were manually highlighted (Fig. 2A), following a general procedure commonly used in biomechanical modeling (Carney, 2016; Bishop, Cuff & Hutchinson, 2021). This was performed for the distal and proximal joint surfaces of each applicable bone, with the final highlighted joint surface saved as a separate digital mesh object in .Obj format.

Once the articular surface was identified and saved as a separate object, an automated shape fitting algorithm in Geomagic was used on the saved shape to identify the joint axis and its position. This algorithm best fits a geometric primitive (Sphere, Cylinder, or Plane) to the previously identified and saved joint shape (Fig. 2B). Based on the elongate nature of the shoulder and the observed joint shapes and orientations of the other joints, cylinders were used for each of the joints. Using a cylinder treats each joint as a hinge-like joint, with the central axis of the cylinder used as the central axis and joint center (marked as the Z axis). Once the cylinder was created in Geomagic that shape was saved as both an .Obj file and as a Vrml1 file, that were later used in the creation of a joint axis locator.

**Creation of Joint Coordinate Systems (JCS’s)**. Once the joint surface and cylinder were saved from Geomagic, those shapes, along with the original bone mesh, were uploaded back into Maya. In Maya, the joint articular surfaces and cylinders were imposed onto each bone, in the same position when created in Geomagic. This results in a series of cylinders at each joint surface, both proximal and distal, for each bone analyzed in the forelimb. Using custom code written for Maya (Gatesy et al., 2022), a set of XYZ axes were fit to the center of each joint cylinder (Fig. 2C). When imported, the Vrml1 file centered the Z axis in the cylinder, representing the central axis of the joint’s movement. The X axis was then re-oriented by hand, along the stationary Z axis, to point proximally and parallel to the length of the bone. This triple axis system resulted in the Z axis representing the axis of flexion-extension (FE), the X axis matching long axis rotation (LAR), and the Y axis representing abduction-adduction (ABAD). These axis systems are known as an anatomical coordinate system (ACS’s); (Grood & Suntay, 1983; Gatesy et al., 2010; Gatesy et al., 2022). Orienting the X axis proximally is opposite to the standard proposed by Manafzadeh & Gatesy (2021), but this was done to better compare this study with previous physical ROM studies.

**Figure 3:**
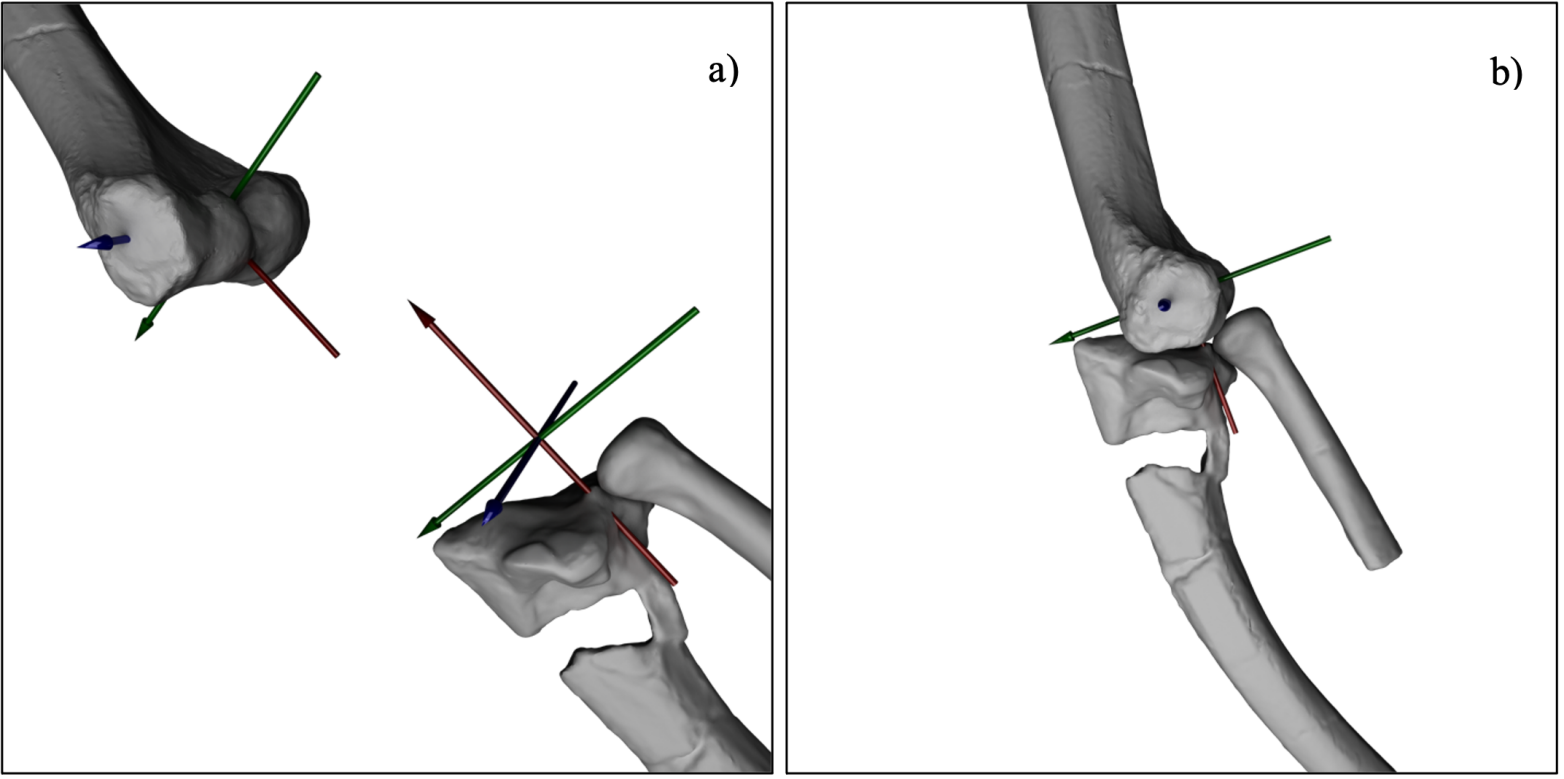
Process for linking two anatomical coordinate systems (ACS’s) into a joint coordinate system (JCS). (A) Two ACS’s with XYZ axes oriented. (B) Completed JCS with both ACS coordinates lying on top of each other.

Each joint is comprised of two ACS’s, one on the proximal, or ‘parent’, bone and one on the distal, or ‘child’, bone (Fig. 3A). Once in place, the child ACS and its associated child bone were reoriented and repositioned using the point and orient constraint function in Maya, so that the child ACS would lie directly on top of the parent ACS (Fig. 3B). This process lines up the X, Y and Z axes, putting both proximal and distal bone segments into alignment and forming a zero pose as well as a joint coordinate system (JCS); (Kambic, Roberts & Gatesy, 2014; Bishop, Cuff & Hutchinson, 2021; Gatesy et al., 2022). In a JCS, the proximal element serves as the ‘parent’ immobile element, and the distal element serves as the ‘child’ mobile element that can be translated and rotated along the three primary axes.

**Creating joints at each JCS**. Once linked into a JCS, 3D rigging joints in Maya were created and placed at each joint center, matching the X, Y and Z axes of the joint to the axes of the proximal parent bone. Joints in 3D rigging and animation are used to animate 3D models, rotating a section of a 3D model about a fixed point, not unlike a traditional armature in stop motion animation (Autodesk.com). Once in place, each bone was parented to the joints and bones underneath in the hierarchy, for example the humerus was parented to the elbow joint, the elbow to the radius and ulna, *etc*. The exact movement of the joint, and by extension the JCS could then be measured in Euler degrees directly in software.

### Zero position adjustment and fine tuning

**Figure 4:**
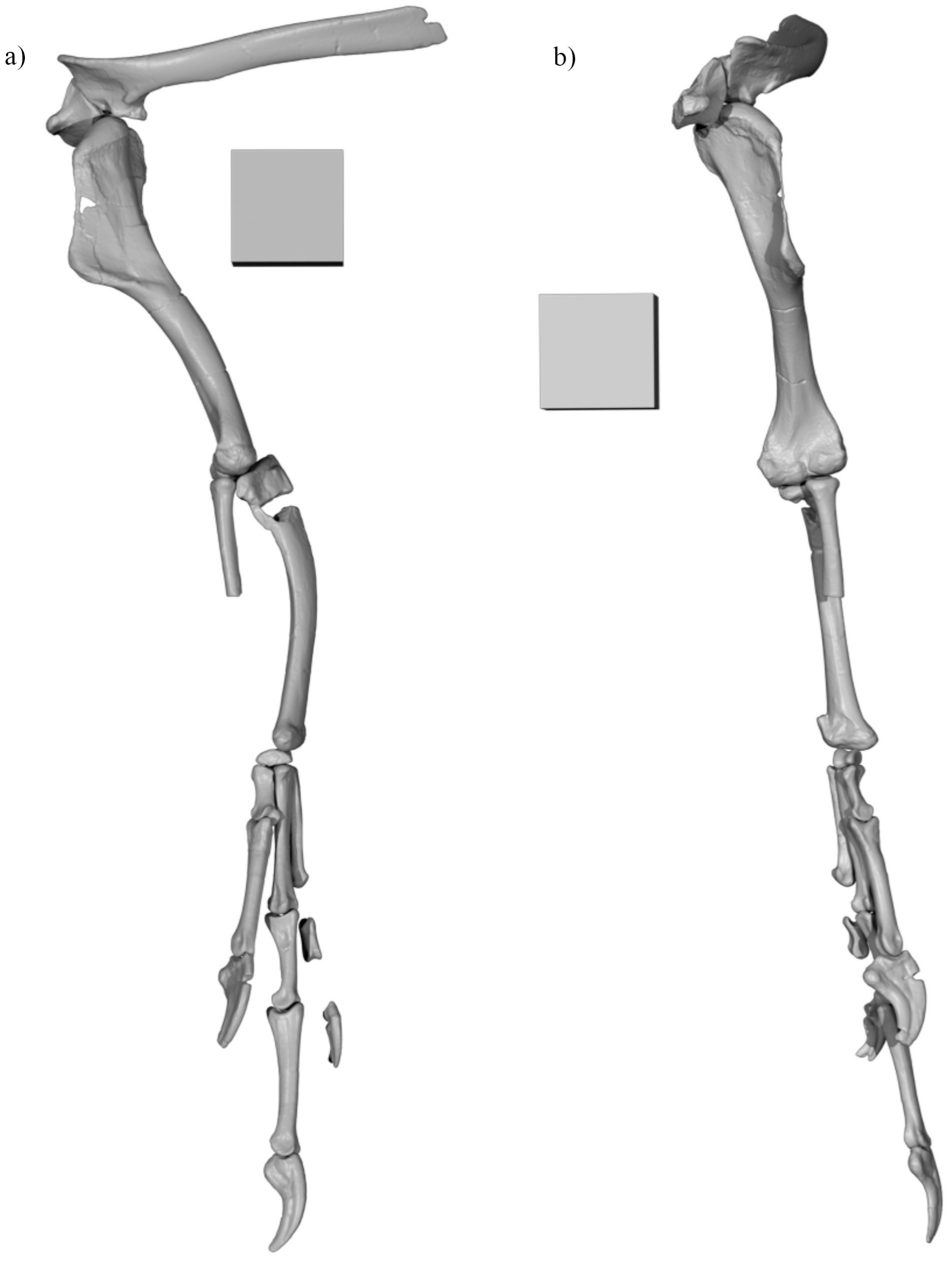
Zero position of the *Troodon* forelimb at lateral (A) and anterior (B) views. Scale cube is five cm.

When initially set up, each joint is placed in its ‘zero position’, where the joint measures ‘zero’ degrees for each of the X, Y and Z axes, as displayed in Fig. 4. The placement of this position was determined by the initial location the X, Y and Z axes. Since the Z axes was largely predetermined by the fit cylinder shape, the positioning of the X axis has the greatest effect on the final joint. Different digital ROM studies have had different methodologies regarding the placement of the X axis, and by extension the zero position of each joint (Bishop, Cuff & Hutchinson, 2021; Gatesy et al., 2022). For this project, the ‘zero position’ was selected by positioning the X axis down the long axis of each bone, pointing proximally, as described above. When all the JCS’s are linked together this results in the X axes linking into a straight line, with the long axis of each element parallel to one another and pointing proximally (Fig. 3B). Given that some bony components were incomplete, the long axis of each element was picked by hand, using the elongated shape of each element as a guide. This zero position was chosen to allow comparison with traditional physical manual manipulation studies (White et al., 2015; Senter & Sullivan, 2019; Yu et al., 2022).

Once the X axes were set, certain joints required manual adjustments to ensure that interpenetration of the bone through a standard rotation of the Z axis (FE) was avoided, or that there wasn’t excessive spacing between joint elements. This involved slight translations on the X axis (adding or subtracting distance between joint elements), slight rotations about the X axis (adjusting the angle of the joint), or both. A detailed description of this process, including a table with the exact adjustments applied, is included in the Supplemental Information.

From the completed digital reconstruction, a ROM assessment at each available joint could take place. This study opted to emulate physical ROM, where the spacing of soft tissues was ignored and up to three rotational degrees of freedom are considered, instead of the full six if translations had also been analyzed. Using the physical ROM approach allowed for comparison with recently described troodontid material from Mongolia (Yu et al., 2022). In addition to measuring flexion-extension (FE), this project also measured abduction-adduction (ABAD) and long axis rotation (LAR).

### Range of Motion measurements

Testing ROM in three axes (flexion-extension, abduction-adduction, long axis rotation) instead of the traditional one (flexion and extension only) posed additional complications for data collection. In the past, tables detailing the separate measurements have been applied, as have animations showing the proposed movement (Manafzadeh & Gatesy, 2022). The latest studies use simulations to systematically measure joint movement and use Boolean processes to remove impossible orientations based on interpenetrating bone (Manafzadeh & Padian, 2018; Demuth, Rayfield & Hutchinson, 2020; Manafzadeh & Gatesy, 2021; Manafzadeh, Gatesy & Bhullar, 2024). This can be very important in testing multiple degrees of freedom, as small changes in one axis can have a large effect on another. Applying this detailed work was beyond the scope of the current study, and instead, manual manipulation of each joint through its range of motion at each of the angular degrees of freedom was used, a compromise between the overly simplistic traditional studies and the current standard of automatic modelling.

**Boolean creation in blender**. To help with identifying interpenetration of bone, the entire mesh and joint model was imported into Blender (Blender Foundation, Amsterdam), where Boolean modifiers were added to copies of each bone mesh, as inspired by previous studies (Manafzadeh & Padian, 2018; Manafzadeh & Gatesy, 2022). These copies occupied the same space as the original bone meshes but were programmed to create a colored Boolean mesh when the meshes intersected. This approach differs from the fully automated approach of the Maya methodology. Blender was chosen for this task because of the assistance of T. Trenkle, a blender specialist who assisted with the construction of the measuring system, allowing for a precise, quick, and objective Boolean-based method of data collection.

**Joint measurement characteristics**. Using the Boolean modifiers, the edge of each movement was identified as bone-on-bone contact (*i.e.,*—Boolean creation). For some joints, Boolean creation resulted in joint movements that appeared unrealistically high, or prematurely low. To compare with these results, the edge of the articular surfaces were also used to define the range of movement. This treats the articular surface of the distal bone as if it cannot pass the edge of the articular surface of the proximal bone, as defined by Senter & Sullivan (2019). While identifying bone-on-bone contact was determined objectively (either a colored shape was, or was not, made), identifying the edge of each joint surface was more subjective. Table 1 includes the bone-on-bone results and these joint surface values. The Supplemental Information contains an alternative to Table 1, with just the bone-on-bone values (Table S3).

**Table 1:** *Troodon* forelimb ROM measurements in euler degrees. E, Extension; F, Flexion; AB, Abduction; AD, Adduction.

***Troodon*** ** Left Forelimb ROM Table (Euler-Degrees)**						
**Joint**	**Flexion-Extension Z (FE)**		**Abduction-Adduction Y (ABAD)**		**Long axis Rotation X (LAR)**	
**Shoulder**	E	81.42	AB	^*^10.27	Lateral	0.00
			AD	23.84	Medial	17.57
			**Total ROM**	**34**.**11**	**Total ROM**	**17**.**57**
	Mid (zero)	0	AB	15.33	Lateral	12.28
			AD	18.58	Medial	13.32
			**Total ROM**	**33**.**91**	**Total ROM**	**25**.**60**
	F	^*^85.97	AB	20.99	Lateral	0.00
			AD	0.00	Medial	50.72
	**Total ROM**	**167.39**	**Total ROM**	**20**.**99**	**Total ROM**	**50**.**72**
**Elbow**	E	25.05	AB	2.92	Lateral	0.00
			AD	0.01	Medial	6.45
			**Total ROM**	**2**.**93**	**Total ROM**	**6**.**45**
	Mid (zero)	0	AB	5.75	Lateral	10.98
			AD	8.38	Medial	18.96
			**Total ROM**	**14**.**13**	**Total ROM**	**29**.**94**
	F	116.54	AB	^*^9.04	Lateral	9.79
			AD	^*^10.21	Medial	^*^31.02
	**Total ROM**	**141.59**	**Total ROM**	**19**.**25**	**Total ROM**	**40**.**81**
**Phalanx I-1**	E	^*^25.00	AB	0.00	Lateral	6.69
			AD	16.49	Medial	0.00
			**Total ROM**	**16**.**49**	**Total ROM**	**6**.**69**
	Mid (zero)	0	AB	^*^26.00	Lateral	6.37
			AD	12.99	Medial	4.76
			**Total ROM**	**38**.**99**	**Total ROM**	**11**.**13**
	F	42.48	AB	0.00	Lateral	0.00
			AD	15.32	Medial	20.68
	**Total ROM**	**67.48**	**Total ROM**	**15**.**32**	**Total ROM**	**20**.**68**
**Ungual Phalanx I-2**	E	^*^28.79	AB	6.34	Lateral	20.09
			AD	2.84	Medial	12.69
			**Total ROM**	**9**.**18**	**Total ROM**	**32**.**78**
	Mid (zero)	0	AB	12.52	Lateral	8.39
			AD	4.05	Medial	25.96
			**Total ROM**	**16**.**57**	**Total ROM**	**34**.**35**
	F	^*^65.69	AB	27.49	Lateral	29.07
			AD	9.66	Medial	13.38
	**Total ROM**	**94.48**	**Total ROM**	**37**.**15**	**Total ROM**	**42**.**45**
**Phalanx II-1**	E	^*^35.50	AB	5.41	Lateral	18.61
			AD	15.15	Medial	20.86
			**Total ROM**	**20**.**56**	**Total ROM**	**39**.**47**
	Mid (zero)	0	AB	7.59	Lateral	15.25
			AD	10.44	Medial	16.39
			**Total ROM**	**18**.**03**	**Total ROM**	**31**.**64**
	F	^*^47.69	AB	5.89	Lateral	15.77
			AD	1.57	Medial	12.95
	**Total ROM**	**83.19**	**Total ROM**	**7**.**46**	**Total ROM**	**28**.**72**
**Phalanx II-2**	E	^*^29.38	AB	12.85	Lateral	2.16
			AD	1.70	Medial	14.82
			**Total ROM**	**14**.**55**	**Total ROM**	**16**.**98**
	Mid (zero)	0	AB	15.71	Lateral	14.64
			AD	16.57	Medial	20.71
			**Total ROM**	**32**.**28**	**Total ROM**	**35**.**35**
	F	^*^60.10	AB	6.40	Lateral	28.51
			AD	24.65	Medial	7.11
	**Total ROM**	**89.48**	**Total ROM**	**31**.**05**	**Total ROM**	**35**.**62**
**Ungual Phalanx II-3**	E	^*^36.24	AB	7.20	Lateral	15.50
			AD	7.53	Medial	12.51
			**Total ROM**	**14**.**73**	**Total ROM**	**28**.**01**
	Mid (zero)	0	AB	3.05	Lateral	12.40
			AD	9.03	Medial	4.76
			**Total ROM**	**12**.**08**	**Total ROM**	**17**.**16**
	F	65.56	AB	0.00	Lateral	0.00
			AD	1.16	Medial	1.47
	**Total ROM**	**101.80**	**Total ROM**	**1**.**16**	**Total ROM**	**1**.**47**

**Notes.**

* Values determined by visually, not bone-on-bone contact.

Bold values represent the total angular movement of joint range of motion values, whether flexion + extension, adduction + abduction or long axis rotation (lateral + medial).

**Measurement process**. Each element was first measured at maximum flexion and extension on the Z axis, also noting the resting position (zero position). For each of these three Z axis positions, the maximum and minimum values for angular range of motion along the X axis (representing long axis rotation) and the Y axis (representing abduction-adduction) were then independently measured. This results in three sets of independent X and Y axis values, measured at each of the corresponding flexed, extended and zero positions. This is a simplified assessment of movement that excludes translational degrees of freedom, as well as interactions between long axis rotation and abduction-adduction, due to required coding that was beyond the scope of the research. This approach was chosen to provide a good overview of potential movement, including not only flexion and extension but the previously mentioned abduction-adduction and long axis rotation, and allows for comparison with traditional physical manipulation studies.

In order to visualize the movement of the reconstructed forelimb through the flexion and extension determined, a skeletal rig was created in Maya. This rig animates the modeled joints together through a full excursion of the extension and flexion values and has been made available in the Supplemental Information for review.

**Physical measurement of 3D printed bones**. Throughout this process 3D printed copies of the forelimb bones were used to help check the digital reconstruction results for selected joints. Using modeling clay to hold bones together, the joint angles were measured with a protractor and results recorded to the closest whole degree. The physical copies have the added benefit of being easier to manipulate and do not interpenetrate like digital copies do. This made for quick reference to double-check joints, especially when movements were not as obvious on the digital model. The physical models were used to help confirm the ROM for all the manual joints in digits I and II but were less helpful with more complicated joints like the shoulder and elbow, which had more than two bones interacting.

## Results

**Figure 5:**
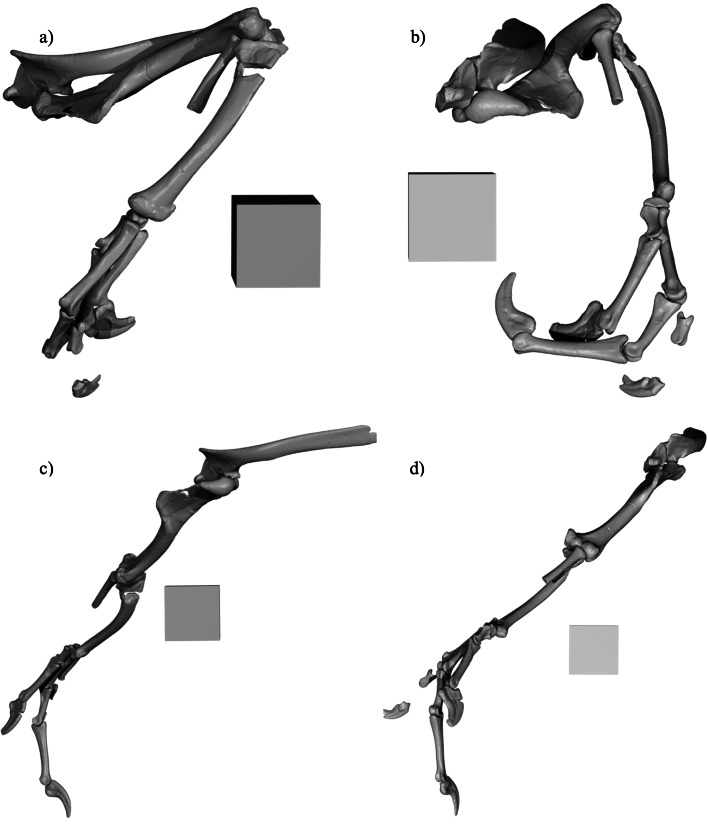
Lateral (A, C) and anterior (B, D) views of the *Troodon* forelimb at flexion (A, B), and extension (C, D) positions. Scale cube is five cm.

**Joint range of motion**. In total, the range of motion (ROM) in seven joints for *T. formosus* was analyzed for this study. Joints analyzed include the shoulder (scapula-coracoid and humerus), elbow (humerus and ulna-radius), and all phalangeal joints from digits I and II, including the ungual joints. All joints were measured in three degrees of freedom, represented by the X, Y and Z axes of the JCS’s. Movement about the Z axis represents flexion and extension (FE) and was recorded at three reference positions: maximum flexion (Figs. 5A, 5B, maximum extension (Figs. 5C, 5D) and the zero pose (Figs. 4A, 4B). From each FE reference position, the maximum angular values for the other two axes were measured: abduction and adduction (ABAD), represented by the Y axis, and long axis rotation (LAR), represented by the X axis (Manafzadeh & Gatesy, 2022). ABAD and LAR were independently measured for each of the FE reference frames. Consistent with findings from other dromaeosaurid and troodontid manual ROM studies (Senter, 2006a; Yu et al., 2022), almost all the forelimb joints analyzed for *T. formosus* had higher ranges of flexion than extension. ROM results for each joint are presented in detail in Table 1. Joints of interest and general patterns are discussed below.

**Figure 6:**
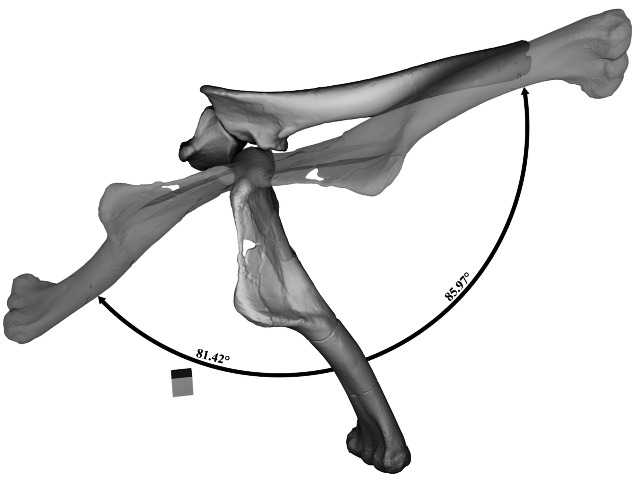
Flexion extension (FE) range of motion for the *Troodon* shoulder. Scale cube is one cm.

**Shoulder**. The shoulder was found to have a total measured ROM of 167 degrees (flexion of 85.97 degrees, and extension of 81.42 degrees). Unlike other joints, flexion and extension were nearly equal, with the flexion angle higher by ∼5 degrees (Fig. 6). The total angular movement (abduction plus adduction) at all three reference positions averaged 29.67 degrees. Long axis rotation (lateral plus medial) across all three positions averaged 31.30, but there were certain poses where maximum LAR appeared to dislocate the humerus from the glenoid, so is unlikely to reflect realistic *in vivo* mobility.

**Figure 7:**
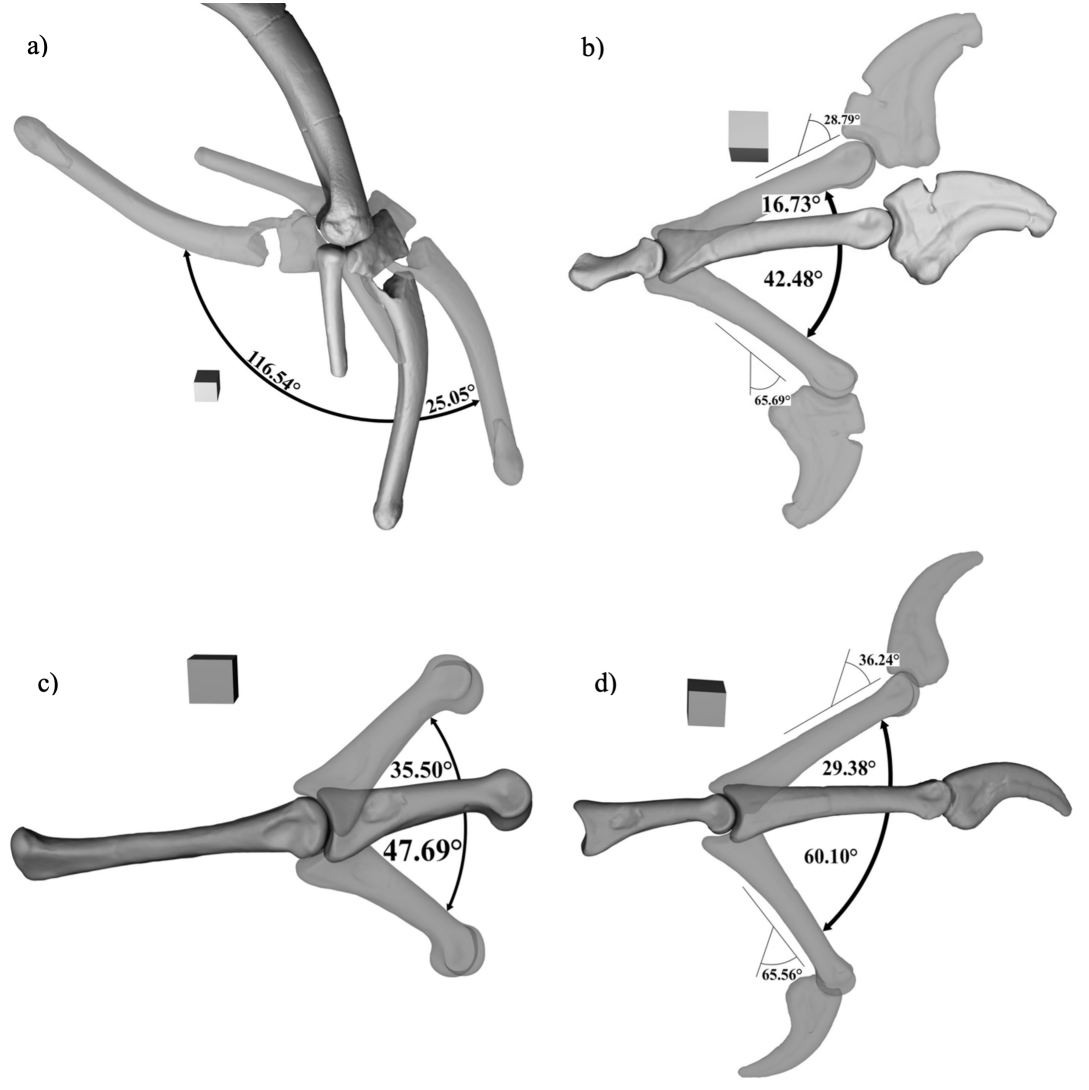
Flexion extension (FE) range of motion for the (A) Elbow, (B) Digit I, (C) Phalanx II-1, (D) Phalanges II-2 and II-3. Scale cube is one cm.

**Elbow**. The elbow’s maximum FE ROM (flexion plus extension) was measured at 141.59 degrees, with significantly higher flexion than extension (Fig. 7A). ABAD and LAR increased as the flexion angle increased.

**Figure 8:**
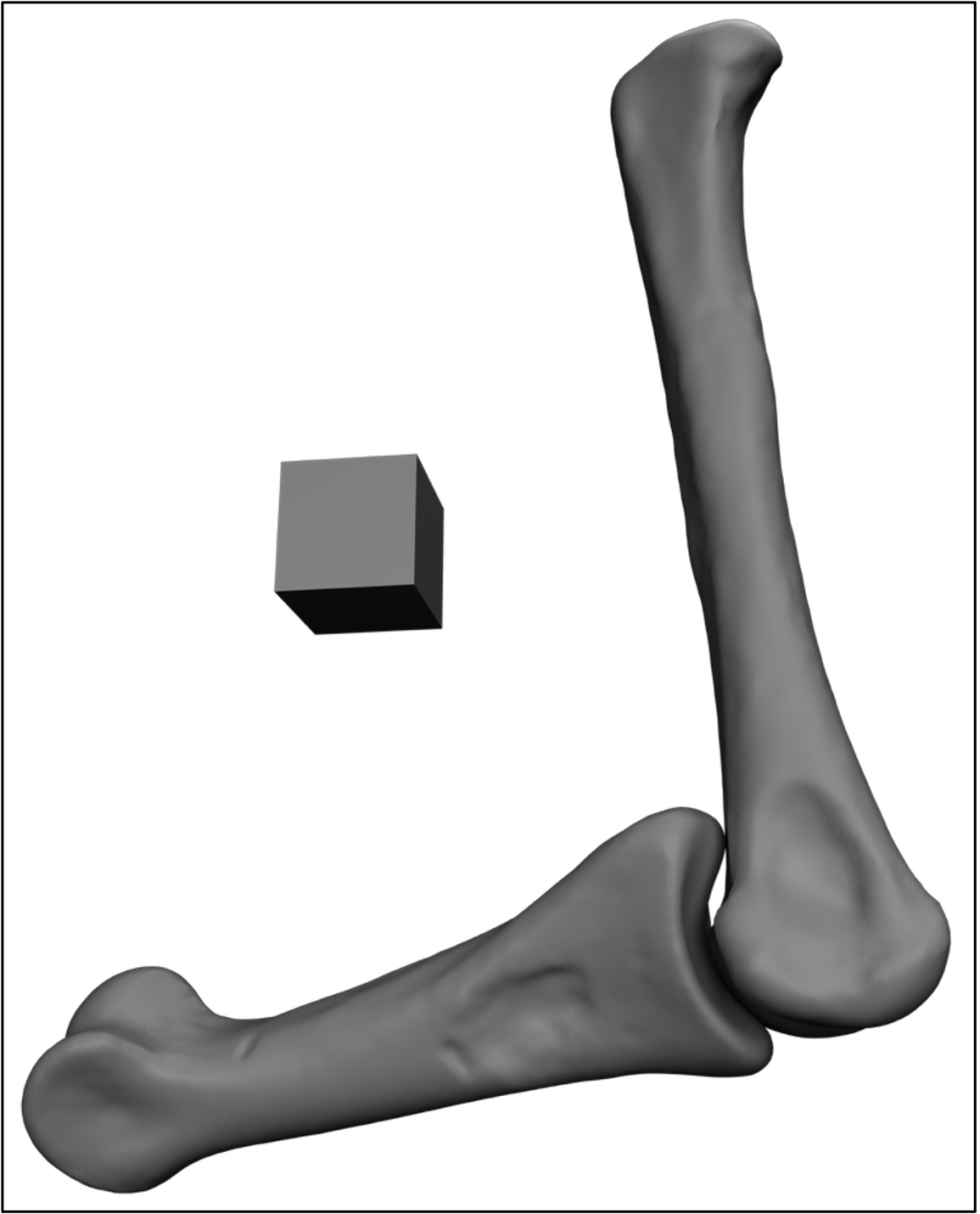
Example of a maximum-flexion manus joint. Note the phalanx appears to be disarticulated at this angle. Scale cube is one cm.

**Figure 9:**
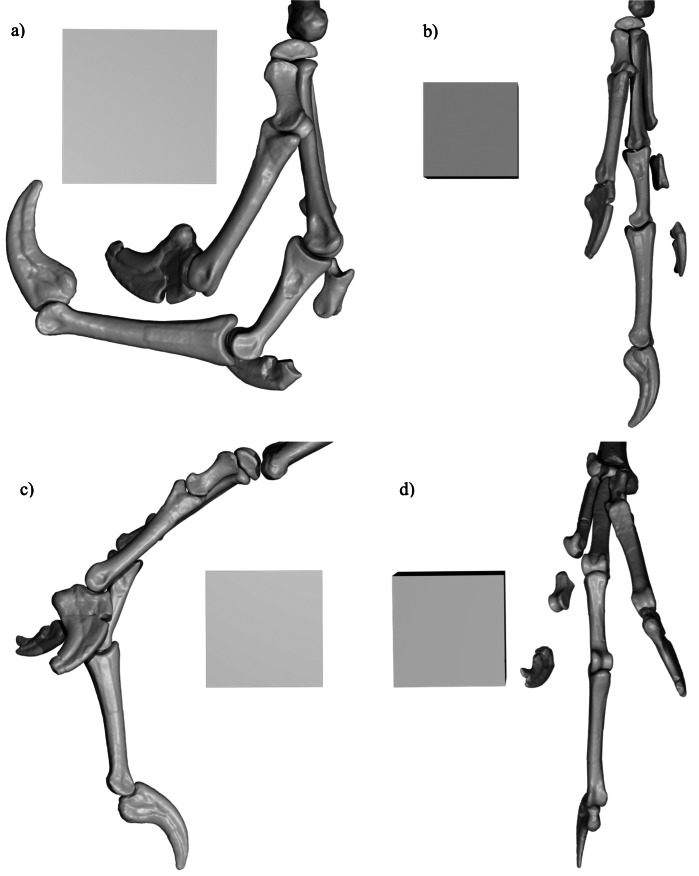
Closeup of *Troodon* manus in (A) flexion, (B) zero, and (C, D) extension positions. Scale cube is five cm.

**Manual joints**. The phalangeal joints follow a similar set of patterns in their ROM, and so will be discussed together here (Figs. 7B, 7C, 7D). Every phalangeal joint had its maximum flexion and extension angle determined by the visual overlap of the proximal and distal articular surfaces, as utilized by physical manipulation studies (Carpenter, 2002; Kobayashi & Barsbold, 2005; Senter & Robins, 2005; Senter, 2006a; Senter, 2006b; White et al., 2015; Senter & Sullivan, 2019; Yu et al., 2022). Bone on bone contact for these joints was deemed too excessive, as it appeared to dislocate the joints in most cases (Fig. 8). Those angles were not used for ROM estimates or hypothesis testing, instead being recorded in the Supplemental Information. The range of flexion was higher than extension for all phalangeal joints analyzed (Fig. 9). However, both ungual joints showed consistently higher ROM than the other phalangeal joints, with an average FE ROM (flexion plus extension) of 98 degrees for ungual joints and 77 degrees for phalangeal joints. The maximum allowed adduction for phalanx I-1 was interrupted by metacarpal II, so it is not likely to represent realistic ROM; instead, more conservative measurements were also taken and recorded in Table 1.

As with other joints, the ROM values for phalanx I-1 were checked with the physical manipulation and measurement of 3D printed models. However, physical manipulations were able to reach much higher maximum extension and abduction angles when compared to the digital model. These results were deemed more realistic, and as such are used in Table 1. ROM measurements for the other phalanx joints confirmed *via* physical measurements corroborated the digital results, so no further changes were necessary.

## Discussion

### Forelimb morphology

**Realistic forelimb resting pose**. The forelimb elements and joints in this study form the most complete reconstruction of a *T. formosus* forelimb that has been made to date. The ‘zero-position’ of this model makes for easier joint measurements but does not reflect a realistic orientation that *T. formosus* would use to rest its forelimb. Figure 1 is a more realistic representation of a *T. formosus* resting orientation. This pose was based off work from Senter & Robins (2015) and was made by orienting each joint to match these findings, within the ROM limits determined in this study (Table 1). The wrist position and placement for the floating elements from digit III could not be based off joint systems and instead were based on complete forelimb elements from related species, especially *Deinonychus* and *Bambiraptor* (Ostrom, 1969; Gishlick, 2002; Senter, 2006a).

**Figure 10:**
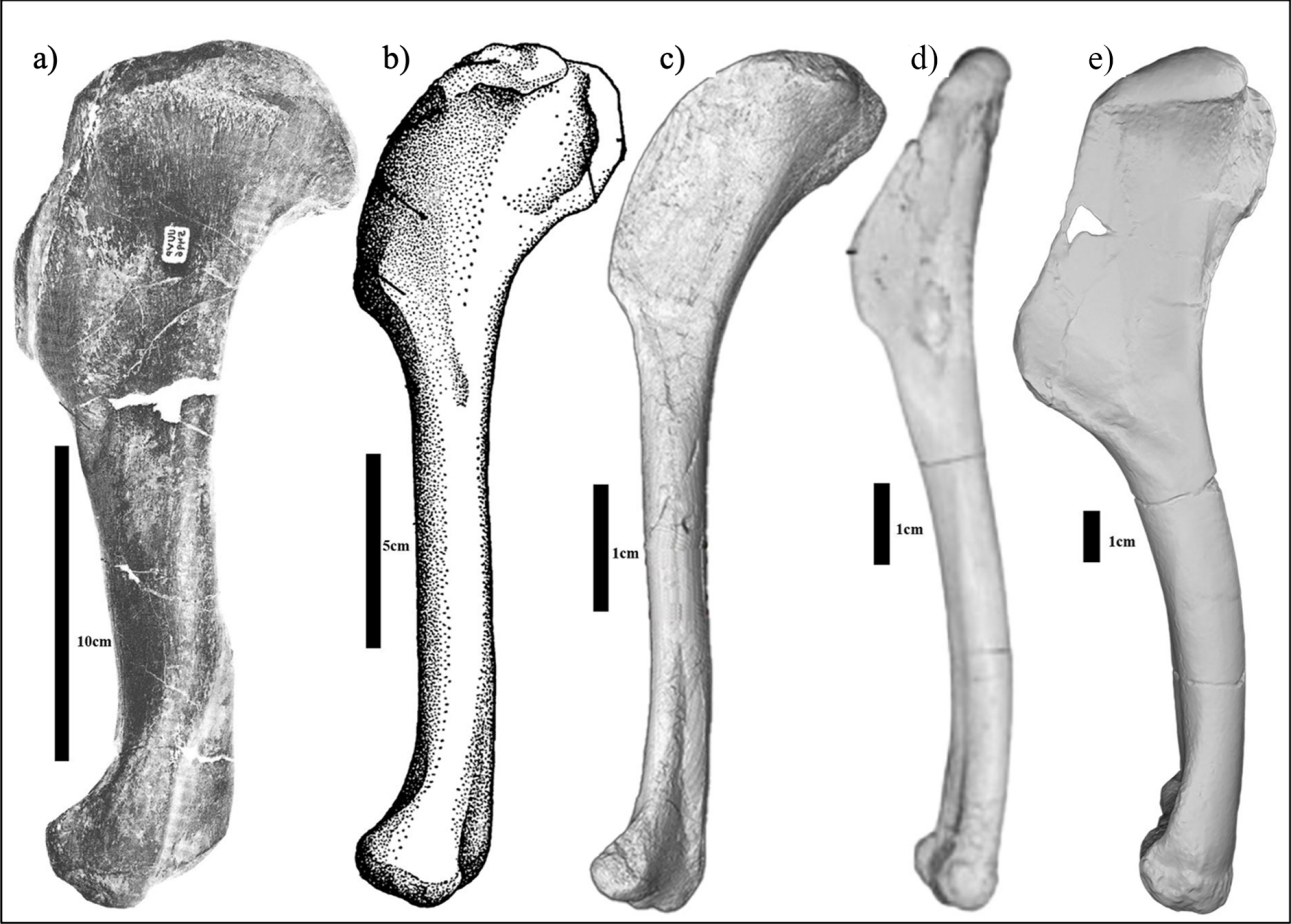
Humerus comparison comparing (A) Allosaurus (Madsen, 1976), (B) Deinonychus, (Ostrom, 1969), (C) Archaeopteryx (Voeten et al., 2018), (D) Gobivenator (Tsuihiji et al., 2014), (E) *Troodon* (this study).

**Humerus mechanical properties**. The forelimb of *T. formosus* (Fig. 1) is robust and stout, especially when compared within Troodontidae and especially for the humerus. Unlike most other paravian species, *T. formosus’s* stocky humerus has a deltopectoral crest (DPC) that extends nearly half the length of the shaft. This contrasts sharply with related paravian species like *Deinonychus, Archaeopteryx, Gobivenator* and *Mei*, which all have a DPC roughly a quarter the length of the humeral shaft (Ostrom, 1969; Gao et al., 2012; Tsuihiji et al., 2014; Voeten et al., 2018). Looking outside of Paraves, the *T. formosus* humerus is more similar to those of larger theropods, like *Allosaurus* (Madsen, 1976); Fig. 10.

Numerous studies have detailed the muscular connections of the DPC, including the connection of the pectoralis, coracobrachialis, and supracoracoideus muscle systems (Jasinoski, Russell & Currie, 2006; Burch, 2014). Generally, these muscles act on the humerus *via* the DPC. The position of the DPC has a tremendous effect on the mechanical leverage that the muscles can produce. The humerus operates akin to a type 3 lever, with the fulcrum (humeral head) acted on by muscle attachments in the DPC (force) that result in movement at the distal humerus (load), (Kent, 2006). Long humeri with short DPC’s, as in *Deinonychus* and *Bambiraptor*, work like a lever with the force located close to the fulcrum. This mechanically produces a long movement arm, which excels at moving quickly, but sacrifices maximum torque. All else being equal, shorter humeri with longer DPC’s, like that of *T. formosus*, perform in an opposite fashion, producing slower movements with higher maximum torque. For example, in living birds a shorter DPC is associated with active flapping flight, whereas a longer DPC is associated with soaring (Serrano & Chiappe, 2017). For non-volant taxa, a higher torque system may indicate a deviation in forelimb use and hunting strategy of *T. formosus* relative to closely related species with shorter DPC’s. For example, this could indicate that *T. formosus* was using its forelimbs to grasp and hold onto potentially struggling or larger prey items, perhaps even making use of its forelimbs as primary hunting tools, similar to megaraptorids (White et al., 2015). In contrast, dromaeosaurid members of Deinonychosauria, including the namesake species *Deinonychus antirrhopus,* are famously thought to rely heavily on the hypertrophied pedal ungual II-3 to aid in prey capture, potentially requiring the forearms less (Fowler et al., 2011).

### Benefits and drawbacks of physical and digital ROM approaches

**Physical ROM**. Through the process of printing physical copies of the digital bones, numerous benefits were observed. It was less obvious in the initial digital reconstruction and scaling, but the scapula and coracoid were initially incorrectly scaled. The scapula was first scaled based on the greatest length of the element, but was incomplete. The awkward scaling was much easier to identify when physical copies of the scapula and coracoid could be compared to the physical copy of the humerus. Once identified, it was an easy fix to rescale the scapula based on its more complete distal width (see Supplemental Information). Additionally, the freedom of manipulating the bones by hand helped to quickly check joint manipulations. The first joint on digit I has a different morphology when compared to the other joints in the manus. This made understanding its preferred orientations and pathways digitally challenging, and in some cases resulted in premature bone-on-bone contact and overlap. Being able to hold metacarpal I and phalanx I-1 by hand to test the ROM provided a much-needed check on the digital system and was able to identify more range than initially assumed.

However, when compared to digital methods, physical ROM can come up short. For example, studies are largely limited to studying complete specimens, with little that can be done with missing and incomplete specimens. Physical methods are also limited in scope, restricted only to measuring skeletal movement, while digital models can add soft tissue to joints, and even muscle (Hutchinson et al., 2005; Burch, 2014; Heers, Rankin & Hutchinson, 2018).

**Digital ROM**. Most of the fossil specimens included in this reconstruction were from multiple individuals at different stages of growth and different sides of the body. For traditional ROM analysis that rely upon whole or associated elements, these specimens could not have been used. Additionally, certain elements were broken or incomplete but were digitally amended, in some cases by combining parts from multiple elements into one. While combining multiple elements from different individuals does carry uncertainties, these techniques allow for the analysis of specimens that would have been previously overlooked.

The digital methods are also not without their drawbacks. The first and largest obstacle to taking up digital ROM methods is the equipment needed. The latest scanning technology and CT scans can create large files that require a specialized, and often dedicated, machine to run. Learning complex digital modeling software introduces an additional time commitment compared to a standard physical ROM assessment.

### Comparative range of motion

**Comparisons with traditional studies**. Table 2 shows a list of forelimb flexion and extension angles for the shoulder, elbow and manual joints for several species of theropods, including *T. formosus*. All the studies included in Table 2 used traditional approaches to measure joint angles, with some using physical bones (Senter, 2006a; Senter & Sullivan, 2019) and others using digital scans of bones (White et al., 2015; Yu et al., 2022). This mix of physical and digital bones can make joint comparisons complicated, since the joint axes can differ. For digital studies, including this one, the axes are fixed and do not change throughout manipulation, while in manual studies they are susceptible to shifting throughout the experiment. To allow for the most comparison with previous traditional physical studies, this study oriented the X axes for each joint down the long axis of each bone pointing proximally, which results in each bone oriented in a straight line for the zero pose (Figs. 4A, 4B). This approach matched closest with the traditional manual ROM axes for the joints, but there was some deviation with the elbow and shoulder.

**Table 2:** Comparative forelimb ROM table.

**Comparative Forelimb ROM Chart, Modifed from Yu et al. (2022) and White et al. (2015)**								
**Dinosaur Taxon**Source	** **	**Shoulder**	Elbow^*^	**I-1**	**I-2**	**II-1**	**II-2**	**II-3**
***Troodon formosus*** (MOR 563, 553)	Extension	81.42	25.05	25.00	28.79	35.50	29.38	36.24
	Flexion	85.97	116.54	42.48	65.69	47.69	60.10	65.56
	**Total ROM**	**167.39**	**141.59**	**67.48**	**94.48**	**83.19**	**89.48**	**101.80**
**SDUST-V1042 (Unnamed troodontid)** Yu et al., 2022	Extension	–	–	–	–	–	4	0
	Flexion	–	–	–	∼71	–	32	9
	**Total ROM**	**–**	**–**	**–**	**–**	**–**	**36**	**9**
***Deinonychus antirrhopus***Senter & Parrish, 2005; Senter, 2006a	Extension	–	150	43	4	10	0	11
	Flexion	–	51	49	70	51	75	85
	**Total ROM**	**–**	**99**	**92**	**74**	**61**	**75**	**96**
***Bambiraptor feinbergi***Senter & Parrish, 2005; Senter, 2006a	Extension	33	127, 136	15	0	28	7	6
	Flexion	88	59, 55	51	76	50	70	92
	**Total ROM**	**121**	**68, 81**	**66**	**76**	**78**	**77**	**98**
***Chirostenotes pergracilis*** Senter & Parrish, 2005	Extension	–	–	5	7	24	25, 16	4
	Flexion	–	–	51	62	41	58, 60	52
	**Total ROM**	**–**	**–**	**56**	**69**	**65**	**83, 76**	**56**
***Gallimimus sp.***Senter & Parrish, 2005; Kobayashi & Barsbold, 2005	Extension	–	–	20	0	25	0	0
	Flexion	–	–	33	96	27	33	90
	**Total ROM**	**–**	**–**	**53**	**96**	**52**	**33**	**90**
***Ornitholestes hermanni***Senter & Parrish, 2005; Senter, 2006b	Extension	–	148	29	0	–	–	17
	Flexion	–	53	52	85	–	–	100
	**Total ROM**	**–**	**95**	**81**	**85**	**–**	**–**	**117**
***Harpymimus okladnikovi***Senter & Parrish, 2005; Kobayashi & Barsbold, 2005	Extension	–	–	28	0	19	14	0
	Flexion	–	–	70	97	25	46	90
	**Total ROM**	**–**	**–**	**98**	**97**	**44**	**60**	**90**
***Guanlong wucaii*** Yu, Corwin & Xing, 2015	Extension	–	–	49	10	46	22	28
	Flexion	–	–	31	71	31	72	33
	**Total ROM**	**–**	**–**	**80**	**81**	**77**	**94**	**61**
***Tyrannosaurus rex***Senter & Parrish, 2005; Carpenter & Smith, 2001	Extension	–	–	35	34	–	–	–
	Flexion	–	–	18	22	–	–	–
	**Total ROM**	**–**	**45**	**53**	**56**	**–**	**–**	**–**
***Australovenator wintonensis*** White et al., 2015	Extension	–	144	40	42	38	37	37
	Flexion	–	66	10	80	36	31	73
	**Total ROM**	**–**	**78**	**50**	**122**	**74**	**68**	**110**
***Acrocanthosaurus atokensis*** Senter & Robins, 2005	Extension	24	159	90	0	77	97	0
	Flexion	109	104	35	3	36	70	35
	**Total ROM**	**133**	**55**	**125**	**3**	**113**	**167**	**35**
***Allosaurus fragilis***Senter & Parrish, 2005; Carpenter & Smith, 2001	Extension	–	–	55	–	20	10	0
	Flexion	–	–	19	–	18	63	58
	**Total ROM**	**–**	**62**	**74**	**–**	**38**	**73**	**58**
***Dilophosaurus wetherilli*** Senter & Sullivan, 2019	Extension	–	150	19	28	105	–	18
	Flexion	–	102	16	65	21	–	27
	**Total ROM**	**85**	**48**	**35**	**93**	**126**	**–**	**45**
***Coelophysis bauri*** Senter & Parrish, 2005	Extension	–	–	18	26	17	13	10
	Flexion	–	–	54	40	48	60	50
	**Total ROM**	**–**	**–**	**72**	**66**	**65**	**73**	**60**

**Notes.**

* *Troodon* antebrachium ROM measurment is additive, other studies referenced are subtractive.

– no value reported.

∼ estimated value.

Bold values represent the total angular movement of joint range of motion values, whether flexion + extension, adduction + abduction or long axis rotation (lateral + medial).

The elbow joint ROM in traditional ROM studies is measured by taking the angle created between the humerus and ulna/radius when in maximum flexion and subtracting it from the angle created when the antebrachium is in maximum extension. This ‘subtractive’ approach obviates the need for a ‘zero-position’ when measuring the antebrachium but makes it difficult to compare with the ACS results. This means that the flexion and extension ROM results cannot be compared between this study and the other traditional studies included in Table 2. However, the total ROM for the antebrachium (bolded values in Table 2) between this and other studies can be compared, as both values represent the maximum arc that the ulna and radius take about the humerus.

The shoulder results from this study are the most difficult to compare. The humeral head shows evidence for movements more complicated than a simple hinge joint and is not modeled as a hinge joint in living birds (*e.g.*, Heers et al., 2016). This study attempted to best understand a complicated joint like the shoulder by simplifying it into a subset of positions (flexion, abduction, *etc.*). This is an oversimplification but still provides some insight into potential movement.

**Flexion and extension comparisons**. Yu et al. (2022) acknowledged the trend in generally increasing flexion and decreasing extension when moving closer to paravian and true avialan dinosaurs. *T. formosus* supports this trend generally, showing flexion within a reasonably close range of ∼20 degrees, especially among closely related species, with the exception of the Mongolian troodontid described in Yu et al. (2022) However, *T. formosus* shows a greatly increased range of extension, especially within the manus, when compared to other closely related and well represented species within paravian dinosaurs. The articulated surfaces for the manual bones used in this study are in excellent condition, so taphonomic or other alterations of the bone surface are likely not resulting in this increase in extension. Depending on the placement of the bones in digital space, the digital marionet produced to measure joint angles digitally could result in an overestimation of extension, but manual joint angles were verified with 3D printed copies and matched most joints analyzed. If this increase in extension ROM is valid, it reflects a reversal by *T. formosus* from the previously noted trend. In addition to the humeral anatomy noted above, this may indicate a deviation in hunting strategy or general forelimb utility relative to other members of Paraves. Perhaps *T. formosus* relied more on the forelimbs to dispatch prey relative the hypertrophied pedal ungual II-3, using the higher range of extension to grasp larger prey. This would align closer to the hunting strategies of larger theropods, including *Australovenator*, *Acrocanthosaurus*, *Allosaurus* and *Dilophosaurus* (Carpenter & Smith, 2001; Senter & Parrish, 2005; Senter & Robins, 2005; White et al., 2015; Senter & Sullivan, 2019).

Interestingly, the flexion and extension ROM for the *T. formosus* manus is unexpectedly higher than the Mongolian troodontid manus described in Yu et al. (2022), specifically for digit II. Reviewing the condition of bones described and analyzed, the Mongolian specimens are crushed and, in some cases, altered well beyond the specimens for *T. formosus*. This could be an explanation for the high deviation in ROM. Given the geographic separation of Asian and North American troodontid species, it is plausible that the two groups of troodontids had different manuals morphologies. However, this hypothesis will be difficult to test without more Mongolian and North American forelimb specimens.

### Whole forelimb ROM and hypothesis testing

This reconstruction has allowed for the assembly of a *T. formosus* forelimb, and for the analysis of whole forelimb ROM hypotheses. Senter (2006a) provides a framework for analyzing functional movement hypotheses. These functional hypotheses were modified and are presented in Table 3. The table includes potential actions, and a set of predictions that serve as mobility and ROM requirements for the action in question. Each prediction was checked against either the measured ROM data, or the related 3D forelimb model, and was either falsified or deemed plausible. Since each prediction serves as a prerequisite for the action in question, if any predictions are falsified the entire action is falsified as well. Importantly, since bone-on-bone ROM studies struggle with verifying true-to-life ROM and mobility, the failure to falsify a hypothesis does not necessarily confirm that an action could have been performed by the living animal in question, just that it is possible. Unless marked with an X, the hypotheses in Table 3 have all been deemed plausible and reasonable.

**Table 3:** Hypotheses and prediction table for *troodon* forelimb movement.

**#**	**Hypotheses and Predictions, Modified From Senter (2006a) and Senter (2006b)**	**Falsified?**
**1**	Both hands together can be used to grip and maintain hold upon an object, with palms and/or palmar surfaces of fingers as the primary organs of prehension.	
** **	Prediction 1: The joints of the forelimb permit the palms to face medially.	
** **	Prediction 2: The joints of the forelimb permit the palms to approach each other medially.	
** **	Prediction 3: The animal is capable of supporting itself bipedally.	
**2**	Both hands together can be used to grip and maintain hold upon an object, with claws as the primary organs of prehension.	
** **	Prediction 1–3: Same as those of Hypothesis 1.	
** **	Prediction 4: The unguals are recurved and sharp.	
** **	Prediction 5: The joints of the forelimb permit the unguals to be oriented with their tips toward the object.	
** **	Prediction 6: The unguals are capable of enough flexion to drive their tips into the object.	
**3**	One hand by itself can be used to grip an object by curling the fingers around the object.	X
** **	Prediction 1: The fingers are long enough to wrap at least halfway around the object.	X
** **	Prediction 2: The fingers are capable of enough flexion to press the object against the palm.	X
**4**	One hand by itself can be used to grip an object between the palmar surfaces of opposing fingers.	X
** **	Prediction 1: At least one finger is opposable to at least one other finger.	X for digits I and II
** **	Prediction 2: The object is small enough to grip between the opposable fingers.	
**5**	The hand can be used as a hook, to bring objects closer to the body.	
** **	Prediction 1: The fingers are capable of enough flexion to form an effective hook.	
** **	Prediction 2: The length and joints of the forelimb permit the hand to be brought near the body while the fingers are flexed (that is, they do not constrain the forelimb to an extended position).	
**6**	The forelimb can be used to bring an object to the mouth.	
** **	Prediction 1: The joints of the forelimb permit the forelimb to reach the mouth.	
** **	Prediction 2: At least one of the following supported: Hypotheses 1-5.	
**7**	The forelimbs can be used to perform a display that involves extending the forelimbs and swinging them dorsoventrally, above the spine.^*^	** **
** **	Prediction 1: The joints of the forelimb allow the limb to be extended.	
** **	Prediction 2: The shoulder joint permits the humerus to swing in a large, transverse arc.	
** **	Prediction 3: The forelimb can be lifted above the spine dorsally.^*^	
**8**	The forelimbs can be used to perform a display that involves extending the forelimbs and swinging them craniocaudally.	** **
** **	Prediction 1: The joints of the forelimb allow the limb to be extended.	
** **	Prediction 2: The shoulder joint permits the humerus to swing in a large, parasagittal arc.	
**9**	The forelimbs can be used to clutch objects to the chest.	
** **	Prediction 1: At least one of the following is supported: Hypotheses 3–7.	
** **	Prediction 2: The humerus can be retracted so that the elbow is level with the ventral surface of the torso.	
** **	Prediction 3: The joints of the forelimb allow the elbow to flex when the humerus is retracted.	
** **	Prediction 4: If the antebrachia are long, the elbow can flex to a strongly acute angle.	
** **	Prediction 5: The animal is capable of supporting itself bipedally.	

**Notes.**

* Hypothesis or prediction modified from Senter (2006a) and Senter (2006b).

**Single and double handed apprehension**. Of particular interest to this study was the potential for single and double handed apprehension of objects, especially eggs. Eggs found from the Two Medicine Formation containing *T. formosus* embryos have been described in detail (Varricchio, Horner & Jackson, 2002), with the dimensions of the eggs well known (Varricchio et al., 2013). The dimensions of the *T. formosus* eggs described in literature were compared to the reconstructed adult manus prepared for this study and manual grasping was assessed for viability according to the hypotheses laid out in Senter (2006a). *T. formosus* eggs are oblong and taper at the base, with the best-preserved example, MOR 299, measured at 138 mm tall and a maximum width of 67 mm (Varricchio et al., 2013). The minimum measured distance across the manus when fully flexed was approximately 79 mm and involved the ungual phalanx from digit I nearly overlapping with digit II (Fig. 9A). The addition of digit III, as well as soft tissue and keratin may add additional support and cushion, but within the bounds of this study the one-handed grasping of its own eggs by *T. formosus* cannot be confirmed. Two handed apprehension of eggs and other objects, the manipulation of its eggs without grasping (*i.e.,* rolling) and without the use of its hands, remains plausible.

**Digit opposability and one-handed grasping**. Based on the mobility of digits I and II, the hypothesis that *T. formosus* could pick up objects the same size and shape as its own eggs with one hand was falsified, as was the potential for digit opposability. However, the flexion movement of digit I brings it towards the midline of the manus, a pattern that was described from analysis of the manual ROM for *Bambiraptor* (Senter, 2006a). In his study, Senter was able to show that digit I approached digit III at maximum flexion when digit II was out of the way. If *T. formosus* follows the same pattern, it may indicate the potential for more flexibility in one-handed grasping than was confirmed by this study.

## Areas of uncertainty

**Scaling**. Assuming a perfectly isometric scaling scheme has undoubtedly introduced a degree of uncertainty to the data. Until a more detailed study of inter-forelimb growth that accounts for ontogeny, this was a necessary simplification. In addition, the techniques required to scale each bone into a representative forelimb varied slightly from element to element. Depending on element completion and association with other elements scaling became more complicated and introduced more uncertainty. As such, not every element used in this reconstruction can be held at the same level of confidence. The scaling process for each element is described in the Supplemental Information, with each elements level of scaling certainty presented in Table S1.

**Ontogeny**. As *Troodon* grew, the morphology of its forelimb bones largely remained the same. However, as was shown by the humerus, adult bones do grow more robust and stocky overtime. Documenting the exact ontogenetic changes in *Troodon* has not yet been published, so how forelimb bones scaled with age is unclear. Bones like the semilunate carpal, scapula and radius were up-scaled more than other elements, but all appear to fit the current reconstruction without issue.

**Shoulder**. The shoulder joint posed a unique challenge, as a complex joint with complex articular surfaces that make it difficult to model. In addition, the shoulder is especially dependent on the constraints of soft tissues, so a bone-on-bone approach likely has a lot of uncertainty (Baier & Gatesy, 2013). Keeping this in mind, the measurements are likely overestimated for this joint.

**Wrist**. The wrist was the only joint outside of digit III that could not be analyzed for this study. Without the distal radius or radiale bone, the exact placement and orientation of the semilunate carpal, and by extension the manus, could not be made by bone orientation alone. Instead, other related species were referenced, especially *Deinonychus*, in orienting the metacarpus and manus into a realistic position (Ostrom, 1969; Gishlick, 2002). Finally, without the wrist, the full medial and lateral ROM of the manus could not be tested in this study and so was left out of hypothesis testing and ROM analysis.

## Conclusions

This study is the first forelimb reconstruction and ROM assessment performed for the theropod dinosaur *T. formosus*. Leveraging digital scanning and 3D printing technology, the disassociated skeletal forelimb elements from multiple individuals of *T. formosus* were assembled into a single forelimb representative of a nearly complete adult arm and hand. Due to missing elements, digit III and the wrist were left out of the ROM study, but the rest of the forelimb was analyzed.

*T. formosus*, like similarly related members of Paraves, shows increased flexion in its forelimb joints relative to extension. However, *T. formosus* shows much higher extension when compared to closely related groups, with ROM totals (flexion plus extension) generally closer with larger more basal theropod relatives, including *Australovenator*, *Acrocanthosaurus*, *Allosaurus* and *Dilophosaurus* (Table 2). Additionally, *T. formosus* has a much stockier forelimb when compared within Paraves and even within other troodontids, more closely resembling the aforementioned basal theropods. This potentially indicates a deviation in hunting strategy or forelimb usage more similar to these larger theropods. Based on ROM results, one-handed grasping of objects, especially eggs, by *T. formosus* has been falsified, but future material could update this result.

More work into the soft tissue spacing in the forelimbs of bipedal theropod dinosaurs would benefit future studies. This study combined the use of digital and physical specimens to aid in the bone-on-bone ROM measurement of *T. formosus’s* forelimb. Further work into the potential overlap of digital and physical methods will likely prove to be beneficial as well.

This study has helped reveal specialization in *T. formosus’s* forelimbs, and potential implications related to hunting and reproductive behavior. Further material collection and study will hopefully round out what we know about *T. formosus* and help explain the peculiarities of this enigmatic theropod.

## Supplemental Information

10.7717/peerj.20987/supp-1 Supplemental Information 1 Supplemental information including the scaling protocol used for each forelimb bone, and the uncertainties present in the study

10.7717/peerj.20987/supp-2 Supplemental Information 2 Certainty table listing each forelimb element used in this study, and the relative certainty in how it was scaled

10.7717/peerj.20987/supp-3 Supplemental Information 3 JCS Modification Table All transformations and rotations made to the joint centers of each joint. Translations are in mm and rotations are in Euler degrees.

10.7717/peerj.20987/supp-4 Supplemental Information 4 Bone on Bone ROM Table Alternative version of Table 1 containing just the objective bone on bone measured joint angles determined in Blender.

10.7717/peerj.20987/supp-5 Supplemental Information 5 *T. formosus* forelimb range of motion animation 3D animation for the *T. formosus* forelimb reconstruction moving through the maximum flexion and extension range determined in this study.

10.7717/peerj.20987/supp-6 Supplemental Information 6 Complete list of 62 MorphoSource specimen DOIs

## Additional Information and Declarations

### Data Availability

The following information was supplied regarding data availability: The data is available at MorphoSource. The complete list of 62 DOIs is available in the Supplemental Files. - https://www.morphosource.org/projects/000723606?locale=en.

## References

[ref-1] Baier DB, Gatesy SM (2013). Three-dimensional skeletal kinematics of the shoulder girdle and forelimb in walking *Alligator*. Journal of Anatomy.

[ref-2] Bishop PJ, Brocklehurst RJ, Pierce SE (2023). Intelligent sampling of high-dimensional joint mobility space for analysis of articular function. Methods in Ecology and Evolution.

[ref-3] Bishop PJ, Cuff AR, Hutchinson JR (2021). How to build a dinosaur: musculoskeletal modeling and simulation of locomotor biomechanics in extinct animals. Paleobiology.

[ref-4] Boekenheide HR (2023). The hind limb ontogeny of troodon formosus. Master’s thesis.

[ref-5] Brocklehurst RJ, Mercado M, Angielczyk KD, Pierce SE (2025). Adaptive landscapes unveil the complex evolutionary path from sprawling to upright forelimb function and posture in mammals. PLOS Biology.

[ref-6] Burch SH (2014). Complete forelimb myology of the basal theropod dinosaur *Tawa hallae* based on a novel robust muscle reconstruction method. Journal of Anatomy.

[ref-7] Carney RM (2016). Evolution of the Archosaurian shoulder joint and the flight stroke of Archaeopteryx. Ecological and evolutionary biology theses and dissertations. Brown Digital Repository.

[ref-8] Carpenter K (2002). Forelimb biomechanics of nonavian theropod dinosaurs in predation. Senckenbergiana Lethaea.

[ref-9] Carpenter K, Smith M (2001). Forelimb osteology and biomechanics of Tyrannosaurus rex. Mesozoic vertebrate life.

[ref-10] Demuth OE, Rayfield EJ, Hutchinson JR (2020). 3D hindlimb joint mobility of the stem-archosaur Euparkeria capensis with implications for postural evolution within Archosauria. Scientific Reports.

[ref-11] Fowler DW, Freedman EA, Scannella JB, Kambic RE (2011). The predatory ecology of deinonychus and the origin of flapping in birds. PLOS ONE.

[ref-12] Freimuth WJ, Varricchio DJ, Brannick AL, Weaver LN, Wilson Mantilla GP (2021). Mammal-bearing gastric pellets potentially attributable to *Troodonformosus* at the cretaceous egg mountain locality, two medicine formation, Montana, USA. Palaeontology.

[ref-13] Galton PM (1971). Manus movements of the coelurosaurian dinosaur Syntarsus and opposability of the theropod hallux. Arnoldia.

[ref-14] Gao C, Morschhauser EM, Varricchio DJ, Liu J, Zhao B (2012). A second soundly sleeping dragon: new anatomical details of the chinese troodontid mei long with implications for phylogeny and taphonomy. PLOS ONE.

[ref-15] Gatesy SM, Baier DB, Jenkins FA, Dial KP (2010). Scientific rotoscoping: a morphology-based method of 3-D motion analysis and visualization. Journal of Experimental Zoology Part A: Ecological Genetics and Physiology.

[ref-16] Gatesy SM, Manafzadeh AR, Bishop PJ, Turner ML, Kambic RE, Cuff AR, Hutchinson JR (2022). A proposed standard for quantifying 3-D hindlimb joint poses in living and extinct archosaurs. Journal of Anatomy.

[ref-17] Gilmore CW (1924). On Troodon validus, an orthopodous dinosaur from the Belly River Cretaceous of Alberta, Canada. Department of Geology, University of Alberta Bulletin.

[ref-18] Gishlick AD (2002). The functional morphology of the forelimb of deinonychus antirrhopus and its importance for the origin of avian flight. Dissertation.

[ref-19] Griffin BW, Martin-Silverstone E, Pêgas RV, Meilak EA, Costa FR, Palmer C, Rayfield EJ (2024). Modelling take-off moment arms in an ornithocheiraean pterosaur. PeerJ.

[ref-20] Grood ES, Suntay WJ (1983). A joint coordinate system for the clinical description of three-dimensional motions: application to the knee. Journal of Biomechanical Engineering.

[ref-21] Heers AM, Baier DB, Jackson BE, Dial KP (2016). Flapping before flight: high resolution, three-dimensional skeletal kinematics of wings and legs during avian development. PLOS ONE.

[ref-22] Heers AM, Rankin JW, Hutchinson JR (2018). Building a bird: musculoskeletal modeling and simulation of wing-assisted incline running during avian ontogeny. Frontiers in Bioengineering and Biotechnology.

[ref-23] Herbst EC, Manafzadeh AR, Hutchinson JR (2022). Multi-joint analysis of pose viability supports the possibility of salamander-like hindlimb configurations in the permian tetrapod *Eryops megacephalus*. Integrative and Comparative Biology.

[ref-24] Hutchinson JR, Anderson FC, Blemker SS, Delp SL (2005). Analysis of hindlimb muscle moment arms in Tyrannosaurus rex using a three-dimensional musculoskeletal computer model: implications for stance, gait, and speed. Paleobiology.

[ref-25] Jasinoski SC, Russell AP, Currie PJ (2006). An integrative phylogenetic and extrapolatory approach to the reconstruction of dromaeosaur (Theropoda: Eumaniraptora) shoulder musculature. Zoological Journal of the Linnean Society.

[ref-26] Kambic RE, Roberts TJ, Gatesy SM (2014). Long-axis rotation: a missing degree of freedom in avian bipedal locomotion. Journal of Experimental Biology.

[ref-27] Kent M (2006). The Oxford dictionary of sports science & medicine.

[ref-28] Kobayashi Y, Barsbold R (2005). Anatomy of Harpymimus okladnikovi Barsbold and Perle 1984 (Dinosauria; Theropoda) of Mongolia. The Carnivorous Dinosaurs.

[ref-29] Leidy J (1860). Extinct Vertebrata from the Judith River and Great Lignite Formations of Nebraska. Transactions of the American Philosophical Society.

[ref-30] Madsen JH (1976). *Allosaurus fragilis*: a revised osteology. Utah Geological Survey Bulletin.

[ref-31] Mallison H (2010). CAD assessment of the posture and range of motion of Kentrosaurus aethiopicus Hennig 1915. Swiss Journal of Geosciences.

[ref-32] Manafzadeh AR, Gatesy SM (2021). Paleobiological reconstructions of articular function require all six degrees of freedom. Journal of Anatomy.

[ref-33] Manafzadeh AR, Gatesy SM (2022). Advances and challenges in paleobiological reconstructions of joint mobility. Integrative and Comparative Biology.

[ref-34] Manafzadeh AR, Gatesy SM, Bhullar B-AS (2024). Articular surface interactions distinguish dinosaurian locomotor joint poses. Nature Communications.

[ref-35] Manafzadeh AR, Padian K (2018). ROM mapping of ligamentous constraints on avian hip mobility: implications for extinct ornithodirans. Proceedings of the Royal Society B: Biological Sciences.

[ref-36] Nopcsa F (1901). Synopsis un Abstammung der Dinosaurier. Földtani Közlöny.

[ref-37] Novas FE, Puerta PF (1997). New evidence concerning avian origins from the Late Cretaceous of Patagonia. Nature.

[ref-38] Ostrom JH (1969). Osteology of Deinonychus antirrhopus, an unusual theropod from the lower cretaceous of Montana. The Peabody Museum Bulletin.

[ref-39] Palma Liberona JA, Soto-Acuña S, Mendez MA, Vargas AO (2019). Assesment and interpretation of negative forelimb allometry in the evolution of non-avian Theropoda. Frontiers in Zoology.

[ref-40] Ramezani J, Beveridge TL, Rogers RR, Eberth DA, Roberts EM (2022). Calibrating the zenith of dinosaur diversity in the campanian of the western interior basin by CA-ID-TIMS U–Pb geochronology. Scientific Reports.

[ref-41] Richards HL, Bishop PJ, Hocking DP, Adams JW, Evans AR (2021). Low elbow mobility indicates unique forelimb posture and function in a giant extinct marsupial. Journal of Anatomy.

[ref-42] Rogers RR, Horner JR, Ramezani J, Roberts EM, Varricchio DJ (2025). Updating the upper cretaceous (Campanian) two medicine formation of Montana: lithostratigraphic revisions, new CA-ID-TIMS U-Pb ages, and a calibrated framework for dinosaur occurrences. Geological Society of America Bulletin.

[ref-43] Rogers RR, Swisher CC, Horner JR (1993). 4° Ar/39Ar age and correlation of the nonmarine Two Medicine Formation (Upper Cretaceous), northwestern Montana, U.S.A. Canadian Journal of Earth Sciences.

[ref-44] Russell DA (1969). A new specimen of *Stenonychosaurus* from the oldman formation (Cretaceous) of Alberta. Canadian Journal of Earth Sciences.

[ref-45] Russell DA, Seguin R (1982). Reconstructions of the small cretaceous theropod *Stenonychosaurus inequalis* and a hypothetical dinosauroid. Syllogeus.

[ref-46] Senter P (2006a). Comparison of forelimb function between *Deinonychus* and *Bambiraptor* (Theropoda: Dromaeosauridae). Journal of Vertebrate Paleontology.

[ref-47] Senter P (2006b). Forelimb function in ornitholestes hermanni osborn (Dinosauria, Theropoda): forelimb function in a cretaceous theropod. Palaeontology.

[ref-48] Senter P, Parrish MJ (2005). Functional analysis of the hands of the theropod dinosaur Chirostenotes pergracilis: evidence for an unusual paleoecological role. PaleoBios.

[ref-49] Senter P, Robins JH (2005). Range of motion in the forelimb of the theropod dinosaur *Acrocanthosaurus atokensis*, and implications for predatory behaviour. Journal of Zoology.

[ref-50] Senter P, Robins JH (2015). Resting orientations of dinosaur scapulae and forelimbs: a numerical analysis, with implications for reconstructions and museum mounts. PLOS ONE.

[ref-51] Senter P, Sullivan C (2019). Forelimbs of the theropod dinosaur Dilophosaurus wetherilli: range of motion, influence of paleopathology and soft tissues, and description of a distal carpal bone. Palaeontologia Electronica.

[ref-52] Sereno PC (1994). The pectoral girdle and forelimb of the basal theropod herrerasaurus ischigualastensis. Journal of Vertebrate Paleontology.

[ref-53] Serrano FJ, Chiappe LM (2017). Aerodynamic modelling of a Cretaceous bird reveals thermal soaring capabilities during early avian evolution. Journal of the Royal Society Interface.

[ref-54] Sternberg CM (1945). Pachycephalosauridae proposed for dome-headed dinosaurs, Stegoceras lambei, n. Sp. described. Journal of Paleontology.

[ref-55] Tsuihiji T, Barsbold R, Watabe M, Tsogtbaatar K, Chinzorig T, Fujiyama Y, Suzuki S (2014). An exquisitely preserved troodontid theropod with new information on the palatal structure from the Upper Cretaceous of Mongolia. Naturwissenschaften.

[ref-56] Van der Reest AJ, Currie PJ (2017). Troodontids (Theropoda) from the Dinosaur Park Formation, Alberta, with a description of a unique new taxon: implications for deinonychosaur diversity in North America. Canadian Journal of Earth Sciences.

[ref-57] Varricchio DJ (1995). Taphonomy of Jack’s Birthday site, a diverse dinosaur bonebed from the upper cretaceous two medicine formation of Montana. Palaeogeography, Palaeoclimatology, Palaeoecology.

[ref-58] Varricchio DJ, Hogan JD, Freimuth WJ (2021). Revisiting Russell’s troodontid: autecology, physiology, and speculative tool use ^1^. Canadian Journal of Earth Sciences.

[ref-59] Varricchio DJ, Hogan JD, Gardner JD (2025). Troodontid specimens from the cretaceous two medicine formation of Montana (USA) and the validity of *Troodon formosus*. Journal of Paleontology.

[ref-60] Varricchio DJ, Horner JR, Jackson FD (2002). Embryos and eggs for the Cretaceous theropod dinosaur *Troodon formosus*. Journal of Vertebrate Paleontology.

[ref-61] Varricchio DJ, Jackson F, Borkowski JJ, Horner JR (1997). Nest and egg clutches of the dinosaur Troodon formosus and the evolution of avian reproductive traits. Nature.

[ref-62] Varricchio DJ, Jackson FD, Jackson RA, Zelenitsky DK (2013). Porosity and water vapor conductance of two *Troodon formosus* eggs: an assessment of incubation strategy in a maniraptoran dinosaur. Paleobiology.

[ref-63] Varricchio DJ, Jin X, Jackson FD (2015). Lay, brood, repeat: nest reuse and site fidelity in ecologic time for two Cretaceous troodontid dinosaurs. Journal of Vertebrate Paleontology.

[ref-64] Voeten DFAE, Cubo J, De Margerie E, Röper M, Beyrand V, Bureš S, Tafforeau P, Sanchez S (2018). Wing bone geometry reveals active flight in Archaeopteryx. Nature Communications.

[ref-65] White MA, Bell PR, Cook AG, Barnes DG, Tischler TR, Bassam BJ, Elliott DA (2015). Forearm range of motion in Australovenator wintonensis (Theropoda, Megaraptoridae). PLOS ONE.

[ref-66] Xu X, Han F, Zhao Q (2014). Homologies and homeotic transformation of the theropod ‘semilunate’ carpal. Scientific Reports.

[ref-67] Yu Y, Corwin S, Xing X (2015). Three-dimensional modeling of the manual digits of the theropod dinosaur Guanlong, with a preliminary functional analysis. Acta Palaeontologica Sinica.

[ref-68] Yu D, Pei R, Yin Y-L, Zhou C-F (2022). The morphology and function of the manual digits of a troodontid from the Yixian Formation of western Liaoning, China. Historical Biology.

