# Supplementary material for: First forelimb reconstruction and range of motion assessment of the Late Cretaceous dinosaur Troodon formosus
Source: PeerJ. 2026 Jul 16;14:e20987. doi: 10.7717/peerj.20987 (PMC13380887; doi:10.7717/peerj.20987)
Supplement: Supplemental Information 1 [file peerj-14-20987-s001.docx]

# **SUPPLEMENTARY MATERIAL**

# **Specimen Scaling Details**

**Scapula**. Since neither the scapulae from MOR 553 nor 563 preserved the greatest length, the scaling was based on their greatest width instead. This was measured as the distance between the most anterodorsal point of the acromial process and the most posterodorsal section of the glenoid fossa, which was preserved in both MOR 553 and 563. This greatest width measurement from MOR 563 was divided by the greatest distal width of the humerus from MOR 563, defined as the maximum width across the humeral distal condyles. Dividing both measurements created a proportion of the scapula width by the humerus width. This proportion was multiplied by the humerus distal width from MOR 553, providing a distal width estimate that was used to scale the MOR 553 scapula, increasing it 1.618 times its original size. The humerus distal width was chosen to scale the scapula, instead of the greatest humeral length, to better match the scapular distal widths from both MOR 553 and 563. Considering the element completion for the distal widths of both the scapulae and humeri, the scaling for this specimen is more certain (see the forelimb modification and certainty table in the supplementary materials). Once scaled, the scapula was mirrored to match the left side material.

**Coracoid**. The suture surface connecting the scapula and coracoid were preserved in both MOR 553 specimens, this allowed for a convenient landmark to scale the coracoid to. Using the size of the suture surface from the scaled scapula, the suture surface of the coracoid was scaled in Maya increasing the coracoid 1.046 times its original size, as measured from its greatest length. Once scaled in this fashion the glenoid surfaces of both the scapula and coracoid were examined and determined to be of equal width. Considering that the coracoid was scaled by eye to match the scapula the scaling for the coracoid is considered more certain. The coracoid was also mirrored to match the left side material.

**Humerus**. The two humeri used in this reconstruction come from MOR 553 S, 8-2-91-303 and 553 S, 7-19-0-96 (certainty table). The deltopectoral crest (DPC) preserved in 91-303 is nearly complete. All the other mature humeri preserved from Jack’s Birthday Site, including 0-96, have DPC’s that are fractured or damaged. In 91-303 there is only a small hole in one portion of the DPC, but it preserves the entirety of its length. The DPC is relatively long, covering almost half the total length of the humerus in *Troodon*. The distal condyles preserved in 91-303, however, are partially preserved, lacking the definition present in other similarly sized humeri from MOR 553. To address this the condyles in the digital copy of 91-303 were manually modified. Using the more complete condyles from 0-96 as a guide, and the mesh modeling tools in Maya, the condyles in 91-303 were shaped to better match a more complete set. The final version of the humerus is a composite. It’s important to note that the modifications made do not alter the greatest length, or greatest width of 91-303, outside of the condyles the rest is original.

**Ulna**. The ulna was scaled using the greatest humeral lengths from MOR 553 and 563. The greatest length recorded for the ulna from MOR 563 was divided by the greatest humeral length from MOR 563, creating a scaling proportion. This proportion was multiplied by the greatest length of the MOR 553 humerus, providing the estimated length for the 553 ulna, which was digitally scaled to 1.246 times its original size in Maya. Even though it was broken, the re-assembled greatest length of the MOR 553 ulna, along with the length from MOR 563, were in good condition. As such the scaling for this specimen is more certain.

**Radius**. Considering the partial nature of both radii the scaling process used the greatest shaft width, instead of length or distal width. The greatest shaft width from the MOR 563 radius was divided by the greatest humeral length from MOR 563, creating a scaling proportion. This scaling proportion was multiplied by the MOR 553 humeral length, providing an estimate for the MOR 553 radius shaft width, which was scaled in Maya 1.427 times its original size. Since the distal width was not available for both radii it was decided to stick to the humeral length as a scaling factor to remain consistent with the rest of the forelimb elements. Considering the condition of the MOR 563 and 553 radius specimens this specimen is considered least certain.

**Semilunate Carpal**. Due to a lack of semilunate carpals preserved for MOR 563, and the good condition of the distal articular surface in MOR 553, this specimen was scaled to visually fit the proximal condyle for metacarpals I and II. To achieve this the semilunate carpal was scaled in Maya up 1.548 times its original size. This resulted in a good visual, and physical fit, with the digital and 3-D printed copies confirming a snug connection between the metacarpals and the semilunate. Since the scaling for the semilunate carpal is based largely off other metacarpals, especially metacarpal-II, its scaling certainty is less certain.

**Metacarpal-I**. The MOR 563 metacarpal-I, like the other MOR 563 metacarpals, is incomplete and required more steps to scale. The first step involved estimating the greatest length for the MOR 563 metacarpal-I specimen by dividing the maximum proximal width of the MOR 563 and 553 specimens, creating a scaling proportion. This proportion was multiplied by the greatest length of the complete MOR 553 metacarpal-I, giving an estimate for the greatest length for the MOR 563 metacarpal-I. This value was then divided by the greatest length of the MOR 563 humerus, creating a second scaling proportion. This final proportion was multiplied by the greatest length from the MOR 553 mature humerus, resulting in an estimated adult length for the MOR 553 metacarpal-I. Ultimately metacarpal-I was scaled down 1.009 times its original size, which in addition to metacarpal-III and phalanx II-1, are the only elements reduced in size. Considering the complicated process required to scale metacarpal-I, the scaling is less certain.

**Metacarpal-II**. Like metacarpal-I, metacarpal-II was scaled using the two-step scaling process outlined above. The first step involved dividing the distal widths of the MOR 563 and 553 metacarpal-II specimens, resulting in a proportion. That proportion was then multiplied by the greatest length of the complete MOR 553 metacarpal-II specimen, giving an estimated MOR 563 metacarpal-II greatest length. This length estimate was then divided by the MOR 563 humerus greatest length, creating a second proportion. This proportion was finally multiplied by the mature MOR 553 humeral length, resulting in an estimated MOR 553 metacarpal-II length. The length of the MOR 553 metacarpal-II was increased in Maya 1.402 times from its original size. Since metacarpal-II followed a similar scaling process as metacarpal-I, both have been defined as less certain.

**Metacarpal-III**. Following the other two metacarpals, the two-step scaling process was used to estimate the greatest length for metacarpal-III. As before, the distal widths from the MOR 563 and MOR 553 metacarpal-III specimens were used to create the first proportion. This proportion was multiplied by the greatest length from the complete MOR 553 metacarpal-III, giving an estimate for the greatest length of the MOR 563 metacarpal-III. This greatest length was divided by the humeral greatest length from MOR 563, creating the second proportion. Finally, this proportion was multiplied by the MOR 553 humeral greatest length, providing the estimate for the MOR 553 metacarpal-III greatest length. Metacarpal-III was decreased 1.0003 times its original size. Following the other two metacarpals, metacarpal-III is less certain.

**Phalanx I-1**. The phalanx I-1 used in the study is a composite element containing portions from a MOR 553 and MOR 563 phalanx, which required a different scaling approach. Each specimen was scaled by dividing the greatest length of the MOR 563 phalanx I-1 by the greatest humeral length from MOR 563, creating a scaling proportion. This proportion was multiplied by the greatest humeral length from MOR 553, creating an estimated total length for phalanx I-1. This length was used to separately scale both the MOR 553 and 563 phalanx specimens. Once scaled, phalanx I-1 from MOR 553 was mirrored to match MOR 563. Next, the MOR 563 and 553 phalanges I-1 were spliced off at the same length down the shaft. The proximal section from MOR 563 was then attached to the distal section of MOR 553, creating one composite phalanx. Total length and width were preserved as best as possible, ultimately approximating a complete phalange. Prior to splicing, the MOR 553 phalanx I-1 was increased in size 1.652 times its original size and the MOR 563 specimen was increased by 1.672 times. Due to the composite nature of the element, the scaling for phalanx I-1 is defined as less certain.

**Ungual Phalanx I-2**. Ungual I-2, like all the other unguals and the humerus, were not scaled. It was determined that scaling was not necessary based on the visual fit that each ungual had with their respective phalanges. This was verified via visual inspection with both the digital and physical copies of the bone, confirming a reasonable fit.

**Phalanx II-1**. Phalanx II-1 was scaled by dividing the greatest length from the MOR 563 II-1 specimen with the humeral greatest length from MOR 563, creating a scaling proportion. This proportion was multiplied by the greatest humeral length of MOR 553, creating an estimated total length for MOR 553 phalanx II-1. Of important note, the MOR 563 phalanx II-1 specimen was partial in length, and its total length was estimated, so the size determined may be off. Considering this incompleteness, phalanx II-1’s scaling is least certain. Phalanx II-1 was decreased 1.034 times its original size.

**Phalanx II-2**. Phalanx II-2 was again scaled using the greatest length of the MOR 563 II-2 phalanx divided by the MOR 563 humeral greatest length and multiplying that result by the greatest length of the MOR 553 humerus. This final measurement was used to increase the MOR 553 phalanx II-2 1.373 times its original size. Considering the relative completion of the elements used, the scaling certainty for phalanx II-2 is defined as more certain.

**Ungual Phalanx II-3**. Like the other unguals, phalanx II-3 was not scaled.

**Phalanx III-2**. Phalanx III-2 was again scaled by dividing the MOR 563 phalanx III-2 greatest length by the MOR 563 humeral greatest length and multiplying the resulting proportion by the humeral length of MOR 553. When applied in Maya this resulted in increasing the phalanx 1.275 times its original size. Importantly, the MOR 563 phalanx III-2 was partial, and its total length estimated, so the estimated value of the MOR 553 phalanx may be off. Considering the estimated length of the scaled element, the scaling is least certain.

**Ungual Phalanx III-4**. Like the other unguals, ungual phalanx III-4 was not scaled.

# **Manual Adjustments to JCS Orientation**

Once in place slight adjustments to the joint coordinate systems (JCS’s) were required to ensure interpenetration of the bone was not occurring prematurely, or that the joint spacing wasn’t too large, with priority given to rotations along the Z axis. This required shifting the joint center, which also moved the bone mesh and all the other distally connected elements linked in a parent-child relationship. Most of the joints were adjusted via translation on the X axis, moving the mesh distally or proximally relative to the proximal mesh, resulting in adding or subtracting joint spacing, respectively. Two joints were adjusted via rotation about the X axis, and in both cases moved dextrally (to the right) relative to their starting point, better centering the joint movement. Only one joint (phalanx II-2) was both translated and rotated relative its original position.

The exact distance in mm and degrees of joint space adjustment is documented in the JCS modifications table included in the supplementary materials. The X, Y and Z values for each joint centra are largely determined by the position of the model in 3D space, however by applying a 3-dimensional distance formula: (${(x_{1}-x_{2})}^{2}+{(y_{1}-y_{2})}^{2}+{(z_{1}-z_{2})}^{2}$), the distance between the original and adjusted point can be determined, with translation away from the proximal bone recorded as a positive value, and translation towards recorded as a negative value. The angular difference is recorded as the difference from the original position and modified joint position orientations, mostly in the X plane.

# **ROM Table with Just Bone-on-Bone Measurements**

Also included in the supplementary materials is an alternative version of Table 1 from the main document. This version of the table includes just the bone-on-bone digital ROM measurements, as opposed to the subjectively picked values. While being more objective, this table includes joint poses that the authors considered too extreme to be viable and were subsequently replaced with more moderate results in Table 1.
