# Supplementary material for: First forelimb reconstruction and range of motion assessment of the Late Cretaceous dinosaur Troodon formosus
Source: PeerJ. 2026 Jul 16;14:e20987. doi: 10.7717/peerj.20987 (PMC13380887; doi:10.7717/peerj.20987)
Supplement: Supplemental Information 2 [file peerj-14-20987-s002.docx]

| ***Troodon formosus* Forelimb Specimens** | | | | | | | |
| --- | --- | --- | --- | --- | --- | --- | --- |
| **Museum #** | | **Field #** | **Specimen ID** | **Mirrored** | **Scaling*** | **Scaling Certainty** | **Notes** |
| MOR | 553 S | 7-24-91-196 | Scapula | Yes | Up-scaled | More Certain | Scaled from greatest distal width |
| MOR | 553 S | 2021-C-182 | Coracoid | Yes | Up-scaled | More Certain | Scaled to match scapula |
| MOR | 553 S | 8-2-91-303 | Humerus | Yes | N/A | N/A | Distal condyles modified based on 7.19.0.96 |
| MOR | 553 S | 7-19-0-96 | Humerus | No | N/A | N/A | Used as reference for 8.2.92.303 |
| MOR | 553 S | 8-12-92-219 | Ulna | No | Up-scaled | More Certain | Ulna was broken, had to be merged into one mesh |
| MOR | 553 S | 7-15-91-63 | Radius | No | Up-scaled | Least Certain | Partial radius, only proximal end present. Scaled on maximum width. |
| MOR | 553 S | 7-28-92-105 | Semilunate Carpal | Yes | Up-scaled | Less Certain | Scaled to match metacarpus. Immature element, unfused distal carpal 4. |
| MOR | 553 S | 8-20-92-303 | Metacarpal I | Yes | Down-scaled | Less Certain |  |
| MOR | 553 S | 8-13-92-237 | Metacarpal II | No | Up-scaled | Less Certain |  |
| MOR | 553 S | 7-16-92-1 | Metacarpal III | No | Down-scaled | Less Certain |  |
| MOR | 553 L | 7-25-9-312 | Phalanx I-1 | No | Up-scaled | Less Certain | Distal end spliced onto 563 |
| MOR | 563 |  | Phalanx I-1 | Yes | Up-scaled | Less Certain | Proximal end spliced onto 7.25.9.312. |
| MOR | 553 S | 2021-C-184 | Phalanx I-2 | No | N/A | N/A |  |
| MOR | 553 S | 8-8-92-188 | Phalanx II-1 | No | Down-scaled | Least Certain |  |
| MOR | 553 S | 7-19-9-264 | Phalanx II-2 | No | Up-scaled | More Certain |  |
| MOR | 553 S | 7-9-9-172 | Phalanx II-3 | No | N/A | N/A |  |
| MOR | 553 L | 7-24-88 | Phalanx III-2 | No | Up-scaled | Least Certain |  |
| MOR | 553 S | 6-17-9-6 | Ungual Phalanx III-4 | No | N/A | N/A |  |
| *Specimens scaled from greatest length of element unless specified otherwise. Certainty based on complexity of scaling method. See materials for details. N/A: Specimen not scaled, kept 1:1 to the original fossil material | | | | | | | |
