# Supplementary material for: First forelimb reconstruction and range of motion assessment of the Late Cretaceous dinosaur Troodon formosus
Source: PeerJ. 2026 Jul 16;14:e20987. doi: 10.7717/peerj.20987 (PMC13380887; doi:10.7717/peerj.20987)
Supplement: Supplemental Information 3 — All transformations and rotations made to the joint centers of each joint. Translations are in mm and rotations are in Euler degrees. [file peerj-14-20987-s003.docx]

| ***Troodon* Left Forelimb JCS Modifications** | | | | | | | |
| --- | --- | --- | --- | --- | --- | --- | --- |
| **Joint** |  | **Original Translation** | **Modified Translation** | **Distance (mm)** | **Original Rotation** | **Modified Rotation** | **Difference (Degrees)** |
| **Shoulder** | X | -38.28 | -38.21 | 0.04 | 172.89 | 172.89 | 0.00 |
|  | Y | 404.76 | 404.59 |  | 56.04 | 56.04 | 0.00 |
|  | Z | 8.46 | 8.37 |  | 112.37 | 112.37 | 0.00 |
| **Elbow** | X | 99.62 | 98.69 | -1.39 | 174.70 | 174.70 | 0.00 |
|  | Y | 298.24 | 298.97 |  | 0.25 | 0.25 | 0.00 |
|  | Z | 4.11 | 4.11 |  | 141.85 | 141.85 | 0.00 |
| **Phalanx I-1** | X | -46.95 | -48.14 | 1.57 | 68.56 | 68.56 | 0.00 |
|  | Y | 243.59 | 243.98 |  | -0.44 | -0.44 | 0.00 |
|  | Z | -5.47 | -5.48 |  | -18.05 | -18.05 | 0.00 |
| **Ungual I-2*** | X | -106.76 | -106.76 | 0.00 | 74.22 | 74.22 | 0.00 |
|  | Y | 256.44 | 256.44 |  | 0.41 | 0.41 | 0.00 |
|  | Z | -5.43 | -5.43 |  | -10.65 | -10.65 | 0.00 |
| **Phalanx II-1** | X | -87.74 | -87.74 | 0.00 | 76.91 | 66.91 | 10.00 |
|  | Y | 237.50 | 237.50 |  | 1.04 | 1.04 | 0.001 |
|  | Z | -1.43 | -1.43 |  | 6.82 | 6.82 | 0.00 |
| **Phalanx II-2** | X | -131.35 | -130.14 | -1.49 | 101.02 | 85.02 | 16.00 |
|  | Y | 230.88 | 230.74 |  | -1.30 | -1.30 | 0.00 |
|  | Z | -0.78 | -0.75 |  | -6.44 | -6.44 | 0.00 |
| **Ungual II-3** | X | -195.93 | -196.96 | 1.07 | 88.38 | 88.38 | 0.00 |
|  | Y | 233.02 | 233.02 |  | -0.58 | -0.58 | 0.00 |
|  | Z | -1.42 | -1.43 |  | -0.35 | -0.35 | 0.00 |
| *Joint Not Modified | | | | | | | |
