# Supplementary material for: First forelimb reconstruction and range of motion assessment of the Late Cretaceous dinosaur Troodon formosus
Source: PeerJ. 2026 Jul 16;14:e20987. doi: 10.7717/peerj.20987 (PMC13380887; doi:10.7717/peerj.20987)
Supplement: Supplemental Information 4 — Alternative version of Table 1 containing just the objective bone on bone measured joint angles determined in Blender. [file peerj-14-20987-s004.docx]

| ***Troodon* Left Forelimb ROM Table (Euler-Degrees) - Just Bone-on-Bone Measurements** | | | | | | |
| --- | --- | --- | --- | --- | --- | --- |
| **Joint** | **Flexion-Extension Z (FE)** | | **Abduction-Adduction Y (ABAD)** | | **Long axis Rotation X (LAR)** | |
| **Shoulder** | E | 81.42 | AB | 0.02 | Lateral | 0.00 |
|  |  |  | AD | 23.84 | Medial | 17.57 |
|  |  |  | **Total ROM** | **23.86** | **Total ROM** | **17.57** |
|  | Mid (zero) | 0.00 | AB | 15.33 | Lateral | 12.28 |
|  |  |  | AD | 18.58 | Medial | 13.32 |
|  |  |  | **Total ROM** | **33.91** | **Total ROM** | **25.60** |
|  | F | 55.87 | AB | 20.99 | Lateral | 0.00 |
|  |  |  | AD | 0.00 | Medial | 50.72 |
|  | **Total ROM** | **137.29** | **Total ROM** | **20.99** | **Total ROM** | **50.72** |
| **Elbow** | E | 25.05 | AB | 2.92 | Lateral | 0.00 |
|  |  |  | AD | 0.01 | Medial | 6.45 |
|  |  |  | **Total ROM** | **2.93** | **Total ROM** | **6.45** |
|  | Mid (zero) | 0.00 | AB | 5.75 | Lateral | 10.98 |
|  |  |  | AD | 8.38 | Medial | 18.96 |
|  |  |  | **Total ROM** | **14.13** | **Total ROM** | **29.94** |
|  | F | 116.54 | AB | 0.00 | Lateral | 9.79 |
|  |  |  | AD | 4.50 | Medial | 0.03 |
|  | **Total ROM** | **141.59** | **Total ROM** | **4.50** | **Total ROM** | **9.82** |
| **Phalanx I-1** | E | 16.73 | AB | 0.00 | Lateral | 6.69 |
|  |  |  | AD | 16.49 | Medial | 0.00 |
|  |  |  | **Total ROM** | **16.49** | **Total ROM** | **6.69** |
|  | Mid (zero) | 0.00 | AB | 2.16 | Lateral | 6.37 |
|  |  |  | AD | 12.99 | Medial | 4.76 |
|  |  |  | **Total ROM** | **15.15** | **Total ROM** | **11.13** |
|  | F | 42.48 | AB | 0.00 | Lateral | 0.00 |
|  |  |  | AD | 15.32 | Medial | 20.68 |
|  | **Total ROM** | **59.21** | **Total ROM** | **15.32** | **Total ROM** | **20.68** |
| **Ungual Phalanx I-2** | E | 84.19 | AB | 6.34 | Lateral | 20.09 |
|  |  |  | AD | 2.84 | Medial | 12.69 |
|  |  |  | **Total ROM** | **9.18** | **Total ROM** | **32.78** |
|  | Mid (zero) | 0.00 | AB | 12.52 | Lateral | 8.39 |
|  |  |  | AD | 4.05 | Medial | 25.96 |
|  |  |  | **Total ROM** | **16.57** | **Total ROM** | **34.35** |
|  | F | 83.99 | AB | 27.49 | Lateral | 29.07 |
|  |  |  | AD | 9.66 | Medial | 13.38 |
|  | **Total ROM** | **168.18** | **Total ROM** | **37.15** | **Total ROM** | **42.45** |
| **Phalanx II-1** | E | 88.94 | AB | 5.41 | Lateral | 18.61 |
|  |  |  | AD | 15.15 | Medial | 20.86 |
|  |  |  | **Total ROM** | **20.56** | **Total ROM** | **39.47** |
|  | Mid (zero) | 0.00 | AB | 7.59 | Lateral | 15.25 |
|  |  |  | AD | 10.44 | Medial | 16.39 |
|  |  |  | **Total ROM** | **18.03** | **Total ROM** | **31.64** |
|  | F | 85.99 | AB | 5.89 | Lateral | 15.77 |
|  |  |  | AD | 1.57 | Medial | 12.95 |
|  | **Total ROM** | **174.93** | **Total ROM** | **7.46** | **Total ROM** | **28.72** |
| **Phalanx II-2** | E | 65.28 | AB | 12.85 | Lateral | 2.16 |
|  |  |  | AD | 1.70 | Medial | 14.82 |
|  |  |  | **Total ROM** | **14.55** | **Total ROM** | **16.98** |
|  | Mid (zero) | 0.00 | AB | 15.71 | Lateral | 14.64 |
|  |  |  | AD | 16.57 | Medial | 20.71 |
|  |  |  | **Total ROM** | **32.28** | **Total ROM** | **35.35** |
|  | F | 108.60 | AB | 6.40 | Lateral | 28.51 |
|  |  |  | AD | 24.65 | Medial | 7.11 |
|  | **Total ROM** | **173.88** | **Total ROM** | **31.05** | **Total ROM** | **35.62** |
| **Ungual Phalanx II-3** | E | 52.54 | AB | 7.20 | Lateral | 15.50 |
|  |  |  | AD | 7.53 | Medial | 12.51 |
|  |  |  | **Total ROM** | **14.73** | **Total ROM** | **28.01** |
|  | Mid (zero) | 0.00 | AB | 3.05 | Lateral | 12.40 |
|  |  |  | AD | 9.03 | Medial | 4.76 |
|  |  |  | **Total ROM** | **12.08** | **Total ROM** | **17.16** |
|  | F | 65.56 | AB | 0.00 | Lateral | 0.00 |
|  |  |  | AD | 1.16 | Medial | 1.47 |
|  | **Total ROM** | **118.10** | **Total ROM** | **1.16** | **Total ROM** | **1.47** |
