# Supplementary material for: First forelimb reconstruction and range of motion assessment of the Late Cretaceous dinosaur Troodon formosus
Source: PeerJ. 2026 Jul 16;14:e20987. doi: 10.7717/peerj.20987 (PMC13380887; doi:10.7717/peerj.20987)
Supplement: Supplemental Information 6 [file peerj-14-20987-s006.docx]

**Morphosource DOIs**

1. Original Phalanx III-2 Left [10.17602/M2/M806142](https://doi.org/10.17602/M2/M806142)
2. Original Phalanx II-2 Left [10.17602/M2/M806139](https://doi.org/10.17602/M2/M806139)
3. Original Phalanx II-1 - Left [10.17602/M2/M806136](https://doi.org/10.17602/M2/M806136)
4. Original Metacarpal III - Left [10.17602/M2/M806121](https://doi.org/10.17602/M2/M806121)
5. [Original Metacarpal II - Left 10.17602/M2/M806118](https://doi.org/10.17602/M2/M806118)
6. Original Metacarpal I - Right [10.17602/M2/M806115](https://doi.org/10.17602/M2/M806115)
7. Original Semilunate Carpal Right [10.17602/M2/M806112](https://doi.org/10.17602/M2/M806112)
8. Original Radius - Left [10.17602/M2/M806109](https://doi.org/10.17602/M2/M806109)
9. Original Ulna Shaft Left [10.17602/M2/M806106](https://doi.org/10.17602/M2/M806106)
10. Original Ulna Head Left[10.17602/M2/M806103](https://doi.org/10.17602/M2/M806103)
11. Original Coracoid - Right [10.17602/M2/M806100](https://doi.org/10.17602/M2/M806100)
12. Original Scapula - Right [10.17602/M2/M806097](https://doi.org/10.17602/M2/M806097)
13. Right Humerus [10.17602/M2/M806089](https://doi.org/10.17602/M2/M806089)
14. Humerus Left [10.17602/M2/M803559](https://doi.org/10.17602/M2/M803559)
15. Ungual Phalanx II-3 Cylinder [10.17602/M2/M803552](https://doi.org/10.17602/M2/M803552)
16. Ungual Phalanx II-3 Surface [10.17602/M2/M803549](https://doi.org/10.17602/M2/M803549)
17. Phalanx II-2 Distal Cylinder [10.17602/M2/M803546](https://doi.org/10.17602/M2/M803546)
18. Phalanx II-2 Distal Surface [10.17602/M2/M803541](https://doi.org/10.17602/M2/M803541)
19. Phalanx II-2 Proximal Cylinder [10.17602/M2/M803538](https://doi.org/10.17602/M2/M803538)
20. Phalanx II-2 Proximal Surface  [10.17602/M2/M803535](https://doi.org/10.17602/M2/M803535)
21. Phalanx II-1 Distal Cylinder [10.17602/M2/M803532](https://doi.org/10.17602/M2/M803532)
22. Phalanx II-1 Distal Surface [10.17602/M2/M803529](https://doi.org/10.17602/M2/M803529)
23. Phalanx II-1 Proximal Cylinder [10.17602/M2/M803526](https://doi.org/10.17602/M2/M803526)
24. Phalanx II-1 Proximal Surface [10.17602/M2/M803523](https://doi.org/10.17602/M2/M803523)
25. Metacarpal II cylinder [10.17602/M2/M803520](https://doi.org/10.17602/M2/M803520)
26. Metacarpal II Articular Surface [10.17602/M2/M803517](https://doi.org/10.17602/M2/M803517)
27. Ungual I-2 Cylinder [10.17602/M2/M803514](https://doi.org/10.17602/M2/M803514)
28. Ungual I-2 surface [10.17602/M2/M803511](https://doi.org/10.17602/M2/M803511)
29. Phalanx I-1 Distal Cylinder [10.17602/M2/M803508](https://doi.org/10.17602/M2/M803508)
30. Phalanx I-1 Distal Articular Surface [10.17602/M2/M803505](https://doi.org/10.17602/M2/M803505)
31. Phalanx I-1 proximal cylinder [10.17602/M2/M803502](https://doi.org/10.17602/M2/M803502)
32. Phalanx I-1 Proximal Surface [10.17602/M2/M803499](https://doi.org/10.17602/M2/M803499)
33. Original Phalanx I-1 [10.17602/M2/M803496](https://doi.org/10.17602/M2/M803496)
34. Phalanx I-1 [10.17602/M2/M803493](https://doi.org/10.17602/M2/M803493)
35. Distal metacarpal I cylinder [10.17602/M2/M803490](https://doi.org/10.17602/M2/M803490)
36. Metacarpal I distal articular surface[10.17602/M2/M803487](https://doi.org/10.17602/M2/M803487)
37. Ulna and Radius Proximal Cylinder[10.17602/M2/M803484](https://doi.org/10.17602/M2/M803484)
38. Ulna and Radius proximal surface [10.17602/M2/M803481](https://doi.org/10.17602/M2/M803481)
39. Ulna and Radius Merged [10.17602/M2/M803141](https://doi.org/10.17602/M2/M803141)
40. Distal humeral condyles fit cylinder [10.17602/M2/M803133](https://doi.org/10.17602/M2/M803133)
41. Glenoid Fossa Fit Cylinder [10.17602/M2/M803130](https://doi.org/10.17602/M2/M803130)
42. Scapula and Coracoid Merged [10.17602/M2/M803127](https://doi.org/10.17602/M2/M803127)
43. humeral head fit cylinder [10.17602/M2/M803124](https://doi.org/10.17602/M2/M803124)
44. Distal humeral condyles [10.17602/M2/M800190](https://doi.org/10.17602/M2/M800190)
45. humeral head [10.17602/M2/M800187](https://doi.org/10.17602/M2/M800187)
46. Glenoid Fossa [10.17602/M2/M800183](https://doi.org/10.17602/M2/M800183)
47. Ungual Phalanx III-4 [10.17602/M2/M760161](https://doi.org/10.17602/M2/M760161)
48. Phalanx III-2 [10.17602/M2/M760156](https://doi.org/10.17602/M2/M760156)
49. Ungual Phalanx II-3 [10.17602/M2/M760151](https://doi.org/10.17602/M2/M760151)
50. Phalanx II-2 [10.17602/M2/M760146](https://doi.org/10.17602/M2/M760146)
51. Phalanx II-1 [10.17602/M2/M760140](https://doi.org/10.17602/M2/M760140)
52. Ungual I-2 [10.17602/M2/M760135](https://doi.org/10.17602/M2/M760135)
53. Phalanx I-1 [10.17602/M2/M760130](https://doi.org/10.17602/M2/M760130)
54. Metacarpal III [10.17602/M2/M760115](https://doi.org/10.17602/M2/M760115)
55. Metacarpal II [10.17602/M2/M760110](https://doi.org/10.17602/M2/M760110)
56. Metacarpal I [10.17602/M2/M760100](https://doi.org/10.17602/M2/M760100)
57. Semilunate Carpal [10.17602/M2/M760093](https://doi.org/10.17602/M2/M760093)
58. Radius [10.17602/M2/M760088](https://doi.org/10.17602/M2/M760088)
59. Ulna [10.17602/M2/M759670](https://doi.org/10.17602/M2/M759670)
60. Humerus [10.17602/M2/M759525](https://doi.org/10.17602/M2/M759525)
61. Coracoid [10.17602/M2/M759519](https://doi.org/10.17602/M2/M759519)
62. Scapula [10.17602/M2/M724369](https://doi.org/10.17602/M2/M724369)
